# LC-HRMS Profiling of Paralytic Shellfish Toxins in *Mytilus galloprovincialis* after a *Gymnodinium catenatum* Bloom

**DOI:** 10.3390/md20110680

**Published:** 2022-10-28

**Authors:** Sandra Lage, Pedro Reis Costa, Adelino V. M. Canário, José P. Da Silva

**Affiliations:** 1 Centre of Marine Sciences (CCMAR/CIMAR LA), University of Algarve, Campus de Gambelas, 8005-139 Faro, Portugal; 2 Portuguese Institute for the Sea and Atmosphere (IPMA), Av. Brasília, 1449-006 Lisbon, Portugal

**Keywords:** M-toxins, GC-toxins, *Gymnodinium catenatum*, marine biotoxins, emergent toxins, seafood safety, occurrence data, European waters, shellfish poisoning, LC-HRMS

## Abstract

Saxitoxin and its more than 50 analogues are a group of naturally occurring neurotoxins collectively designated as paralytic shellfish toxins (PSTs). PSTs are toxic to humans and maximum legal limits in seafood have been implemented by regulatory authorities worldwide. In the European Union, monitoring of PSTs is performed using the AOAC Official Method 2005.06, based on liquid chromatography coupled with fluorescence detection (LC- FLD). However, this method has been suggested to not effectively detect the emerging *C*-11 hydroxyl (M-toxins) and benzoate (GC-toxins) analogues, with these analogues currently not being surveyed in monitoring programs. In this study, a liquid chromatography-high resolution mass spectrometry (LC-HRMS) method was used to search for these emerging PSTs in mussels, *Mytilus galloprovincialis,* contaminated following an intense *Gymnodinium catenatum* bloom in the Tagus estuary (Lisbon, Portugal). Five M-toxins (M1, M2, M6, dcM6, and dcM10), but no GC-toxins, were detected in the mussels’ whole-soft body tissue. Moreover, the classical PSTs (C1 to C4, GTX 4 to GTX6, dcGTX1 to dcGTX4, dcSTX, dcNEO, and STX) were also found and comprised the largest fraction of the PSTs’ profile. The presence of unregulated PSTs in edible mussel samples suggests potential seafood safety risks and urges further research to determine the frequency of these analogues in seafood and their contribution to toxicity.

## 1. Introduction

Paralytic shellfish toxins (PSTs) are a group of neurotoxic alkaloids that, in the marine environment, are produced by the bloom-forming dinoflagellates *Gymnodinum catenatum*, *Pyrodinium bahamense,* and *Alexandrium* spp. Filter-feeders, such as mussels, clams, and scallops, ingest the dinoflagellate blooms and accumulate PSTs [1,2,3]. Human consumption of PST-contaminated shellfish leads to a severe and occasionally fatal illness, known as paralytic shellfish poisoning (PSP) syndrome [4]. To protect public health and ensure the quality of seafood, monitoring programs and international trade codes are ongoing worldwide [5]. 

**Scheme 1:**
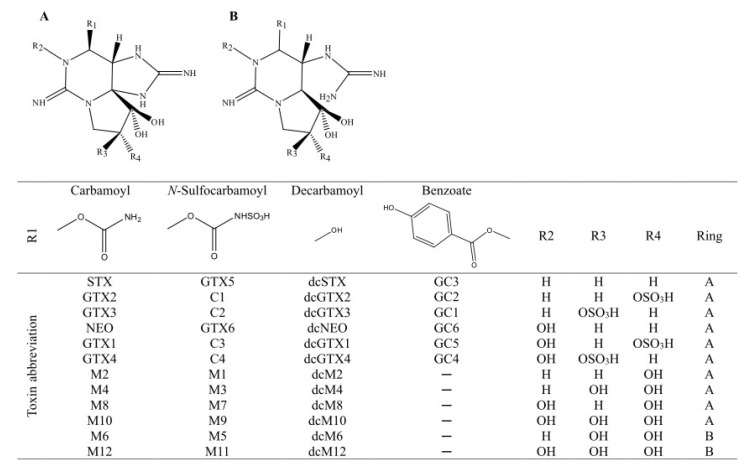
Chemical structures of classical and emerging characterized PST analogues. See Material and Methods Section for toxin abbreviations.

To date, more than 50 PST analogues with a saxitoxin (STX)-based structure have been described [6]. According to their substituent side chains, they are classified into three groups: carbamoyl, *N*-sulfocarbamoyl, and decarbamoyl toxins (Scheme 1) [7]. PST analogues differ in toxicity because of differences in charge and substitutions on the basic STX structure, which modify their binding ability to the Na^+^ channels. Carbamoyl analogues are the most toxic and *N*-sulfocarbamoyl analogues are the least toxic [8,9]. Only a few of these PST analogues are produced by marine dinoflagellates, with the others being formed after enzymatic and chemical reactions in shellfish tissues [2]. 

Advances in mass spectrometry methods led to the discovery of new saxitoxin *C*-11 hydroxyl analogues (Scheme 1). These analogues, named M-toxins, are considered metabolic products of PSTs from shellfish tissues, or resulting from chemical degradation during prolonged storage of shellfish tissues [10,11,12,13,14]. However, trace amounts of M-toxins have also been reported in PST-producing dinoflagellates *Alexandrium* spp. [15,16]. Although the toxicity of M-toxins is still not known, they are proposed to have lower toxicity than the classical PSTs based on structure–activity relationships [10,12,17]. Moreover, a fourth group of PSTs, having a benzoate moiety sidechain (Scheme 1), named GC-toxins, has been identified in several PST-producing *G. catenatum* strains [18]. The toxicity of GC-toxins is also not known. However, a study performed with the rat brain Na^+^ channel revealed strong binding of GC1, GC2, and GC3 to this ion channel, suggesting only slightly lower toxicity than STX, the most potent PST [19]. 

M-toxins and GC-toxins are not regulated and are not adequately detected by the official AOAC 2005.06 reference method for the analysis of PSTs in monitoring programs, which recommends liquid chromatography analysis with fluorescence detection (LC-FLD) [20]. M-toxins give rise to low or residual fluorescent compounds after oxidation, which prevents their detection by LC-FLD [10]. GC-toxins detection by the AOAC 2005.06 method have three issues: (*i*) they are retained in the C18 cartridges used during solid-phase extraction (SPE) sample clean-up as a result of their hydrophobicity; (*ii*) they are partially hydrolysed in strong alkaline media used for samples oxidation; and (*iii*) the fluorescent compounds they generate after oxidation have longer retention times than the other PST analogues ones, thus they might not elute during the official method gradient time [18,21]. Successful detection of these emerging PSTs has been achieved by graphitized carbon SPE cartridges’ clean-up and hydrophilic interaction liquid chromatography-tandem mass spectrometry (HILIC LC-MS/MS) analysis [10,14,15,16,21,22,23,24].

The fact that M-toxins and GC-toxins may pose a food safety risk and are not regularly monitored prompted us to investigate their occurrence and fate in an area where *G. catenatum* is a toxin source [25]. Thus, the main objectives of this study were to determine the profile of classical and emerging PSTs in mussels, *Mytilus galloprovincialis*, contaminated during an intense bloom of *G. catenatum*, using a liquid chromatography-high resolution mass spectrometry (LC-HRMS) method capable of discriminating most PST analogues, both hydrophilic and hydrophobic [26]. 

## 2. Results

### 2.1. Accurate Mass-Extracted Ion Chromatogram (AM-XIC)-Based Quantitation

**Figure 1:**
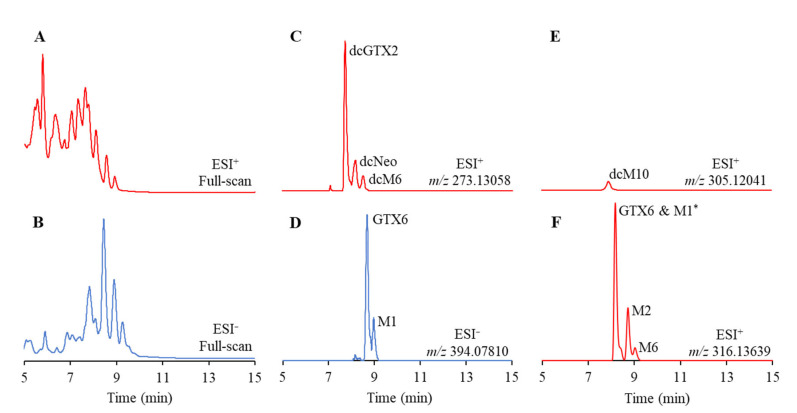
LC-HRMS full-scan positive (ESI^+^) (**A**) and negative (ESI^−^) (**B**) mode profiles of mussels (*M. galloprovincialis*) extract and the correspondent XIC taken at the *m*/*z* values 273.13058 at ESI^+^ (**C**), 394.07810 at (**D**), 305.12041 at ESI^+^ (**E**), and 316.13639 at ESI^+^ (**F**), with a ± 5 ppm window. * GTX6 and M1 signals at *m*/*z* 316.13639 ± 5 ppm at ESI^+^ correspond to *in-*source fragment ions. GTX6 and M1 were quantified under ESI^−^, *m*/*z* 394.07810. Red indicates analysis under ESI^+^ and blue under ESI^−^.

PST analogues’ detection was achieved using graphitized carbon SPE cartridges clean-up and a full-scan HILIC LC-HRMS method (*m*/*z* ranging between 100 and 500), in both positive (ESI^+^) and negative (ESI^−^) modes [26]. PST analogues’ quantitation was performed by generating accurate mass-extracted ion chromatograms (AM-XIC) with the calculated exact masses (5 decimals) of the analogues [M + H]^+^ and [M − H]^−^ ions in the correspondent full-scan LC-HRMS chromatograms and a mass extraction window of ± 5 ppm (Figure 1 and Table 1). Figure 1A,B show the profile obtained under full-scan positive (ESI^+^) and negative (ESI^−^) modes, respectively, and Figure 1C–F show the selected AM-XICs. The absence of signals other than the targeted compounds in the AM-XIC demonstrates the procedure’s selectivity and detection capability [27,28].

To further confirm the emerging PST analogues’ assignments, and in the absence of commercially available certified reference materials (CRMs), their MS^2^ spectra obtained by collision-induced dissociation (CID) and higher-energy collisional dissociation (HCD) (see Supplementary Figures S1–S34) were compared with those reported in previous studies [10,13,14,22]. HCD is known to originate spectra similar to those obtained using triple quadrupole fragmentation [29]. The unequivocal identification of classical PSTs in samples was performed by comparing the observed retention times, exact masses, isotope distributions, and fragmentation spectra obtained by CID and HCD with the same parameters obtained after injection of the authentic standards (CRMs). The CID and HCD MS^2^ spectra were obtained by running the HILIC LC-HRMS under a product ion scan by fragmentation of the ions of each analogue, with the same *m*/*z*, over the entire chromatographic separation and detection (see Section 4).

In Figure 1F, i.e., ESI^+^ *m*/*z* 316.13639 XIC, in addition to M2 and M6 peaks, two additional peaks are observed. After examination of the MS^2^ spectra, the peaks were assigned to GTX6 and M1 ([M + H]^+^ of *m*/*z* 396.09321), which exhibit a loss a SO_3_ group due to *in*-source fragmentation (Supplementary Figures S20, S21, S25 and S26), as previously observed [22]. Note that GTX6 and M1 were quantified under ESI^−^ mode ([M − H]^−^ of *m*/*z* 394.07810) (Figure 1D), where *in*-source fragmentation was not observed. 

Unambiguous identification of GCs was not possible. A small peak was detected at the GC3 exact mass *m*/*z* 377.15679 (ESI^+^), with a retention time of 10.85 min, but had a weak MS^2^ spectra signal, where only the product ion (*m*/*z* 377) and the fragment ions at *m*/*z* 359 and 335 were distinguishable [18,30].

### 2.2. PSTs Profile 

**Figure 2:**
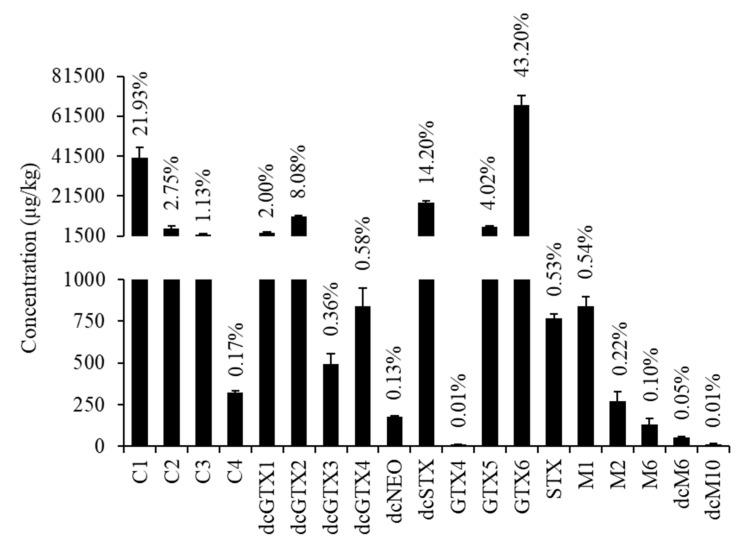
Concentrations of classical and emerging PST analogues in naturally contaminated mussels (*M. galloprovincialis*) extract. Error bars show the standard deviations of three replicate injections. The molar percentage (%) of each toxin is given above the bars. Note: classical GTX1, GTX2, and GTX3 were not detected.

The PST profile of naturally contaminated mussels (*M. galloprovincialis*) during a bloom of *G. catenatum* was composed of five emerging M-toxins (M1, M2, M6, dcM6, and dcM10) and the classical PSTs, *N*-sulfocarbamoyl C1 to C4, GTX5 and GTX6, decarbamoyl, dcGTX1 to dcGTX4, and dcSTX and dcNEO, as well as carbamoyl, STX, and GTX4 (Figure 2).

M-toxins accounted for 0.92% of the total PSTs’ molar fraction. M1 was the M-toxin with the highest concentration, 839 ± 58.6 µg/kg, corresponding to 0.22% of the total molar fraction (Figure 2).

The classical PSTs accounted for 99% of the total PSTs molar fraction (Figure 2). The *N*-sulfocarbamoyl analogues alone represented 73% of the molar fraction (Figure 2). The mussel profile was mainly composed of three classical PSTs (GTX6 > dcSTX > C1), which accounted for 79% of the total PST molar fraction (Figure 2).

### 2.3. PST Toxic Equivalent Quantity

The toxic equivalent quantity (TEQ) of the mussel extract was above the regulatory limit of 800 µg STX diHCl eq/kg, i.e., 33.67 mg STX diHCl equivalent (eq)/kg. Individual PST levels were converted into equivalents of STX using the toxicity equivalency factors (TEFs) proposed by the EFSA panel [17]. For C1, C3, dcGTX1, and dcGTX4, no TEFs have been recommended by EFSA, thus a TEF of 0.01 was used for C1 and C3 and a TEF of 0.5 was used for dcGTX1 and dcGTX4, as previously suggested [31,32]. For M-toxins, no TEFs are available; therefore, they were not accounted for in the PST TEQ calculation.

### 2.4. Matrix Effect and Limits of Detection and Quantification

The evaluation of matrix effects on the quantitative analysis of PSTs in shellfish can strongly influence quantification [33]. Therefore, the matrix effect (ME), limit of detection (LOD), and limit of quantification (LOQ) were determined for each classical PST in a mussel (*M. galloprovincialis*) extract shown to be free from PSTs by LC-HRMS. ME, LOD, and LOQ of dcGTX1, dcGTX4, M-toxins, and GC-toxins could not be determined because of the unavailability of CRMs. 

Most classical PSTs (11 out of 16) had ion suppression, i.e., ME less than 100%, with GTX6, C3, and C4 having the strongest and C1 and GTX3 having the least (Table 2). The other PSTs tested had ion enhancement, i.e., ME above 100% (Table 2). Thus, to account for matrix effects, the PST analogues were quantified with matrix-matched calibration curves in PST-free mussel extract spiked with known concentrations of the classical PSTs (see Section 4). STX had the lowest LOD and LOQ, while GTX2 and GTX5 had the highest (Table 2). 

## 3. Discussion

Five emerging M-toxins (M1, M2, M6, dcM6, and dcM10), but not GC-toxins, were detected in mussels, *M. galloprovincialis*, naturally contaminated during a bloom of *G. catenatum* in the Tagus estuary (Portugal), during the autumn of 2018 (Figure 2). The classical PSTs’ profile observed in the mussels was the typical profile reported for PST-contaminated mussels from Portuguese waters (Figure 2) [34]. The bloom was very intense and extensive, with a maximal cell concentration of 18 × 10^3^ cells L^−1^ and PST TEQs in bivalves exceeding the regulatory limit of 800 µg STX diHCl eq/kg for several weeks. Moreover, a few suspected human PSP cases were reported [20,35,36,37]. Although the PST TEQs and profiles of these mussels were previously evaluated as part of the ongoing Portuguese bivalve monitoring system (SNMB), M-toxins and GC-toxins were not investigated by SNMB [36]. 

In our mussels pooled sample M1 could have been formed from C1/2, the second most abundant PST (Figure 2). This conversion has been suggested in several bivalves, including mussels, based on the reduction of the C1/2 molar fraction in the bivalve in comparison with the levels in the producing dinoflagellates and the concomitant detection of M1 in the bivalve tissues [12,14,34,38]. The formation mechanism appears to start through *O*-desulfation of 11-hydroxysulfate to form M1 [14]. Subsequently, M2, M6, and dcM6 are originated from M1 in some extent. M1 converts into M2 through an *N*-desulfation and into M5 by the opening of the ring at C4 and originating M6 and dcM6 through *N*-desulfonylation (hydrolysis of the *N*-sulfo group) and decarbamoylation (hydrolysis of the carbamate ester), respectively [14]. It has been also proposed that M1 can be originated from GTX5 through hydroxylation at C11 [11] and that M6 can be formed via ring opening at C4 of M4 [13]. The formation of dcM10 in mussel tissues is probably due to the decarbamoylation of M10 [14]. However, the origin of M10 is still not clear. It has been suggested to be an *N*-hydroxy analogue of M4 or to be formed through a *O*-desulfation of GTX4 or from NEO with the formation of an intermediate product [12,14,39]. 

The M-toxins detected here represent only 0.92% of the total PSTs’ molar fraction (Figure 2). It is important to note that the concentration of M-toxins is an estimate based on the equimolar response of another PST analogue CRM (see Section 4). A comparison with other semiquantitative estimates of M-toxins obtained by LC-HRMS using relative molar response factors (RMRs) indicates that the method used here may significantly underestimate M-toxins’ concentration [22]. Previously, it was determined by LC-HRMS that the RMRs for M-toxins were on average 0.17 and 0.0086 for ESI^+^ and ESI^−^, respectively [22]. This underestimation may be due to (*i*) the longer retention times of M-toxins, which elute with a higher percentage of aqueous mobile phase, reducing ESI sensitivity when compared with earlier eluting PST analogues (Figure 1C–F); (*ii*) M-toxins *in-*source fragmentation (Figure 1F) [22]; or (*iii*) matrix interference, i.e., ME of M-toxins different from the ME of the PST analogue used for its semiquantitation (see Section 4). Therefore, there is a need to develop CRMs for M-toxins and to improve their detection and quantification by LC-HRMS.

In our study, unambiguous identification of GC3 was not possible. However, the same mussel sample was previously extracted and analysed using a modified version of the AOAC Official Method 2005.06, and two peaks of the chromatogram were reported to correspond to trace levels of GC1 and GC3 fluorescent compounds after oxidation [35]. High levels of GC-toxins were expected to be detected in these mussels, considering their contamination by a *G. catenatum* bloom, which has a PST profile dominated by GC-toxins [18,21,30,38]. However, because the *G. catenatum* cell concentration was low at the time of sampling [37], it is likely that GC-toxins underwent metabolization and/or elimination in the mussel tissues, leading to undetectable concentrations. In fact, a previous exposure experiment showed that, although GC-toxins account for 10% of the PST molar fraction of mussels fed with *G. catenatum* for 5 days, a concentration reduction of GC1 and GC3 of up to 70 and 20%, respectively, was observed after 24 h of the elimination period [30]. A decrease in GC1 and GC3 in the surf clam (*Spisula solida*) fed with *G. catenatum* was accompanied by an increase in their decarbamoyl analogues, dcGTX2 and dcSTX, respectively, which was suggested to occur because of the activity of carbamoylase [40]. Mussels, *M. galloprovincialis*, lack carbamoylase activity, thus their GC-toxins conversion may have occurred through other unknown processes that require further investigation.

The *N*-sulfocarbamoyl analogues (C1 to C4) and GTX6 comprised 69% of the total PST molar fraction, while the decarbamoyl analogues dcGTX1 to dcGTX4, dcSTX, and dcNeo consisted of 25% (Figure 2), corresponding to a PST TEQ of 33.67 mg STX diHCl equiv/kg. However, the sample analysed right after harvesting had a toxicity level of 38 mg STX diHCl equiv/kg [35]. This slight reduction in TEQ might be due to the mussels’ metabolization and, over time, chemical degradation of classical PST-analogues into M-toxins. In summary, the presence of unregulated PSTs in edible mussel samples suggests potential seafood safety risks and urges further research to determine the frequency of these analogues in seafood and their contribution to toxicity.

## 4. Materials and Methods

### 4.1. Materials

Water and acetonitrile and methanol (LC-MS grade) were acquired from Carlo Erba Reagents (Milan, Italy). Acetic acid, -formic acid, ammonium hydroxide solution 25%, and ammonium formate (LC-MS grade) were purchased from Sigma-Aldrich (Darmstadt, Germany). Certified reference materials (CRMs): N-sulfocarbamoyl gonyautoxin-2 (C1), 40.1 ± 2.4 μg/g; N-sulfocarbamoyl gonyautoxin-3 (C2), 11.5 ± 0.9 μg/g; N-sulfocarbamoyl gonyautoxin-1 (C3), 12.6 ± 0.9 μg/g; N-sulfocarbamoyl gonyautoxin-4 (C4), 3.4 ± 0.3 μg/g; Gonyautoxin-1 (GTX1), 27.3 ± 1.6 μg/g; Gonyautoxin-2 (GTX2), 22.2 ± 1.5 μg/g; Gonyautoxin-3 (GTX3), 8.2 ± 0.6 μg/g; Gonyautoxin-4 (GTX4), 7.3 ± 0.6 μg/g; Gonyautoxin-5 (GTX5), 18.1 ± 1.2 μg/g; Gonyautoxin-6 (GTX6), 10.0 ± 0.5 μg/g; Decarbamoylgonyautoxin-2 (dcGTX2), 35.1 ± 1.9 μg/g; Decarbamoylgonyautoxin-3 (dcGTX3), 8.0 ± 0.9 μg/g; Neosaxitoxin dihydrochloride (NEO), 20.3 ± 1.2 μg/g; Saxitoxin dihydrochloride (STX), 20.3 ± 1.3 μg/g; Decarbamoylneosaxitoxin dihydrochloride (dcNEO), 9.1 ± 0.5 μg/g; and Decarbamoylsaxitoxin dihydrochloride (dcSTX), 19.5 ± 1.7 μg/g, were purchased from CIFGA Laboratories S.A. (Lugo, Spain).

The PST-contaminated mussels (*Mytilus galloprovincialis*), 30 individuals with shell lengths ranging from 6 to 10 cm, were harvested in October 2018 in Porto Brandão (38°40′31.1664″ N; 9°13′13.9044″ W), south of Lisbon, Portugal, during an intense bloom of PST-producing dinoflagellate *Gymnodinium catenatum*. The blank mussels (previously confirmed to have no detectable PSTs by LC-HRMS) were collected in December 2021 in Sagres, Algarve, Portugal.

### 4.2. Sample Preparation

Mussel PST-contaminated and blank samples were extracted according to the protocol of Turner et al. (2015) [24]. Briefly, mussel whole-soft body tissue samples were homogenized in an Ultra-Turrax (T 25 easy clean digital, IKA 107-Werke GmbH & Co. KG, Staufen, Germany); 5 ± 0.1 g of the homogenates was weighed into a 50 mL polypropylene centrifuge tube and 3 mL of acetic acid/water (1:100 *v*/*v*) was added. The mixture was shaken for 3 min on a multi-vortex mixer before being placed into a boiling water bath for 5 min. The samples were cooled to room temperature in running cold water and centrifuged at 2200× *g* for 10 min at 15 °C. A 1 mL supernatant aliquot of the sample extract was transferred into a 1.5 mL polypropylene Eppendorf tube and 5 μL of ammonium hydroxide solution 25% was added. Solid-phase extraction (SPE) clean-up was performed using ENVI-Carb SPE cartridges (250 mg/3 mL volume) (Sigma-Aldrich, Germany). The SPE procedure was performed manually as follows: the cartridges were conditioned with 3 mL of acetonitrile/water/acetic acid (20:80:1 *v*/*v*/*v*), followed by 5 mL water/ammonium hydroxide solution 25% (1000:1 *v*/*v*) eluting to waste; then, 500 µL of sample extracts was loaded onto the conditioned cartridges, washed with 700 μL of Milli-Q water, and eluted to waste; finally, PSTs were eluted with 2 mL of acetonitrile/water/acetic acid (20:80:1 *v*/*v*/*v*) into polypropylene Eppendorf tubes. The eluate was transferred to a polypropylene autosampler vial and diluted with acetonitrile before analysis.

### 4.3. LC-HRMS Analysis

The samples were analyzed by LC-HRMS. Chromatographic separation was carried out using an UltiMate 3000 UHPLC system coupled to an Orbitrap Elite mass spectrometer (Thermo Fisher Scientific Inc., Waltham, MA, USA) equipped with a heated eletrospray ionization source (HESI-II). The PST analogues were separated using an ACQUITY Premier BEH Amide (2.1 × 100 mm, 1.7 μm, Waters, Milford, MA, USA) at 35 °C. Samples were held in the autosampler at 4 °C. The mobile phase was composed of water with 0.1% formic acid and 10 mM ammonium formate (A) and acetonitrile with 0.1% formic acid and 2% 10 mM ammonium formate solution (B). The gradient (in *v*/*v* %) started with 5% of B and increased linearly to 95% in 11 min. This composition was maintained for 1 min and then returned to 5% of B in 1 min and maintained at this composition for 2 min before the next run [26]. The flow rate was 0.3 mL/min and the injection volume was 10 µL. Data were acquired under positive (ESI^+^) and negative (ESI^−^) polarity using the following ionization parameters: spray voltage, 3.8 kV; sheath gas, 40 arbitrary units; auxiliary gas, 10 arbitrary units; heater temperature, 300 °C; capillary temperature, 325 °C; and S-Lenses RF level, 69.06%. The LC-HRMS acquisition was performed under full-scan with the *m*/*z* ranging between 100 and 500. The collision-induced dissociation (CID) and higher-energy collisional dissociation (HCD) MS^2^ spectra of classical and emerging PST analogues were obtained by running the system under a product ion scan by fragmentation of the ions of each analogue, with the same *m*/*z*, over the entire chromatographic separation (LC-HRMS^2^) and detection from 100 to 500 *m*/*z*. The spectra are averages obtained for the corresponding chromatographic peak (Supplementary Figures S1–S34). See the Supplementary Material for information on the energy used for CID and HCD spectra.

### 4.4. PSTs’ Quantitation and Profile Analysis

The LC-HRMS quantitation was performed by generating accurate mass-extracted ion chromatograms (AM-XIC) obtained from full-scan positive and negative profiles using the exact mass (5 decimals) of each PST analogue (Table 1) and a mass extraction window of ±5 ppm [27,28]. Quantification was performed by preparing matrix-matched calibration curves, with five concentration points, in blank mussel extract. Quantitation of STX, NEO, dcSTX, and dcNEO was performed under positive mode (ESI^+^) and quantitation of GTX1 to GTX6, dcGTX2 and dcGTX3, and C1 to C4 was performed under negative (ESI^−^) mode [23,24,26]. Both positive and negative profiles were assessed for the presence of all PST analogues described in Table 1. CRMs for dcGTX1 and dcGTX4 and M-toxins are not commercially available. Thus, dcGTX1 and dcGTX4 were quantified with the matrix-matched calibration curves of dcGTX2 and dcGTX3, respectively. M-toxins were quantitated using the matrix-matched calibration curves of the PST analogues with corresponding chemical structure and expected charge state in solution, i.e., M1 with GTX6, M2 and M6 with STX, and dcM6 and dcM10 with dcSTX. PST analogue concentrations were determined in µmol/kg. PST TEQ, expressed as mg STX diHCl equivalents/kg, was calculated by summing individual PST analogue concentrations and applying TEFs recommended by EFSA [17]. For C1 and C3 and dcGTX1 and dcGTX4, no TEFs were recommended by EFSA, thus a TEF of 0.01 was used for C1 and C3 and a TEF of 0.5 was used for dcGTX1 and dcGTX4, as previously suggested [31,32]. For M-toxins, no TEFs are available; therefore, they were not accounted for in the PST TEQcalculation. 

The limits of detection (LOD) and quantification (LOQ) were calculated from the standard deviations (SDs) obtained after five injections of each blank matrix spiked with the second-lowest concentration (3 × SD and 10 × SD, respectively). The matrix effect (ME) was obtained after three injections of the third-lowest concentration of standard solution and of each blank matrix spiked with this concentration and calculated using the following equation:

(1) $$\mathrm{ME}\left(\%\right)=\frac{B}{A}\times 100$$

where *A* is the average peak area of the standard solution and *B* represents the average peak area in the extract spiked with the same concentration.

## Figures and Tables

**Table 1:** Calculated exact masses of classical and emerging PST analogues’ positive and negative ions.

	PST Analogue	Positive Ion	Calculated Exact Mass	Negative Ion	Calculated Exact Mass
Carbamoyl	STX	[M + H]^+^	300.14148	[M + HCOO]^−^	344.1324
	GTX2	[M + H – SO_3_]^+^	316.13639	[M – H]^−^	394.07810
	GTX3	[M + H]^+^	396.09321		
	NEO	[M + H]^+^	316.13639	[M + HCOO]^−^	360.12732
	GTX1	[M + H – SO_3_]^+^	332.13131	[M – H]^−^	410.07302
	GTX4	[M + H]^+^	412.08812		
	M2	[M + H]^+^	316.13639	[M + HCOO]^−^	360.12732
	M4/M8	[M + H]^+^	332.13131	[M + HCOO]^−^	376.12223
	M10	[M + H]^+^	348.12622	[M + HCOO]^−^	392.11715
	M6	[M + H – H_2_O]^+^	316.13639	[M + HCOO – H_2_O]^−^	360.12732
	M12	[M + H]	350.14187	[M – H]^−^	348.12677
*N*–Sulfocarbamoyl	GTX5	[M + H]^+^	380.09829	[M – H]^−^	378.08319
	C1/C2	[M + H]^+^	476.05002	[M – H]^−^	474.03492
		[M + H – SO_3_]^+^	396.09321		
	GTX6	[M + H]^+^	396.09321	[M – H]^−^	394.07810
	C3/C4	[M + H]^+^	492.04494	[M – H]^−^	490.02983
		[M + H – SO_3_]^+^	412.08812		
	M1	[M + H]^+^	396.09321	[M – H]^−^	394.07810
	M3/M7	[M + H]^+^	412.08812	[M – H]^−^	410.07302
	M9	[M + H]^+^	428.08304	[M – H]^−^	426.34005
	M5	[M + H – H_2_O]^+^	396.09321	[M – H – H_2_O]^−^	394.07810
	M11	[M + H]^+^	430.09869	[M–H]^−^	428.08358
Decarbamoyl	dcSTX	[M + H]^+^	257.13566	[M + HCOO]^−^	301.12659
	dcGTX2	[M + H – SO_3_]^+^	273.13058	[M – H]^−^	351.07229
	dcGTX3	[M + H]^+^	353.08739		
	dcNEO	[M + H]^+^	273.13058	[M + HCOO]^−^	317.12151
	dcGTX1	[M + H – SO_3_]^+^	289.12549	[M – H]^−^	367.06720
	dcGTX4	[M + H]^+^	369.08231		
	dcM2	[M + H]^+^	273.13058	[M + HCOO]^−^	317.12151
	dcM4/dcM8	[M + H]^+^	289.12549	[M + HCOO]^−^	333.11642
	dcM10	[M + H]^+^	305.12041	[M + HCOO]^−^	349.11134
	dcM6	[M + H – H_2_O]^+^	273.13058	[M + HCOO – H_2_O]^−^	317.12151
	dcM12	[M + H]^+^	307.13606	[M + HCOO – H_2_O]^−^	279.14114
Benzoate	GC1/GC2	[M + H]^+^	473.10852		
	GC3	[M + H]^+^	377.15679		
	GC4/GC5	[M + H]^+^	489.10344		
	GC6	[M + H]^+^	393.15171		

**Table 2:** Limits of detection and quantification (LOD and LOQ, µg/kg) and matrix effects with corresponding relative standard deviation (ME ± RSD, %) of classical PST analogues.

PST Analogue	LOD (µg/kg)	LOQ (µg/kg)	ME ± RSD (%)
C1	0.07	0.22	82 ± 1.0
C2	0.02	0.06	61 ± 4.4
C3	0.17	0.56	53 ± 1.8
C4	0.29	0.95	43 ± 1.8
dcGTX2	0.14	0.48	135 ± 14.8
dcGTX3	0.23	0.76	101 ± 3.1
dcNeo	0.08	0.28	73 ± 3.2
dcSTX	0.07	0.22	69 ± 8.6
GTX1	0.28	0.93	120 ± 16.3
GTX2	0.46	1.53	106 ± 9.2
GTX3	0.35	1.17	89 ± 5.3
GTX4	0.07	0.23	105 ± 9.2
GTX5	0.44	1.48	79 ± 1.8
GTX6	0.13	0.43	48 ± 6.3
NEO	0.02	0.08	79 ± 9.3
STX	0.01	0.04	69 ± 6.0

## Data Availability

The data generated in this study is contained within this article and supplementary materials.

## Supplementary Materials

The following supporting information can be downloaded at https://www.mdpi.com/article/10.3390/md20110680/s1. Figure S1. CID MS^2^ spectrum of *m*/*z* 474 (C1,2) in *M. galloprovincialis* at ESI^−^. Energy: 25 arbitrary units; Figure S2. HCD MS^2^ spectrum of *m*/*z* 474 (C1,2) in *M. galloprovincialis* at ESI^−^. Energy: 55 arbitrary units; Figure S3. CID MS2 spectrum of *m*/*z* 490 (C3,4) in *M. galloprovincialis* at ESI^−^. Energy: 18 arbitrary units; Figure S4. HCD MS^2^ spectrum of *m*/*z* 490 (C3,4) in *M. galloprovincialis* at ESI^−^. Energy: 55 arbitrary units; Figure S5. CID MS^2^ spectrum of *m*/*z* 367 (dcGTX1,4) in *M. galloprovincialis* at ESI^−^. Energy: 35 arbitrary units; Figure S6. HCD MS^2^ spectrum of *m*/*z* 367 (dcGTX1,4) in *M. galloprovincialis* at ESI^−^. Energy: 55 arbitrary units; Figure S7. CID MS^2^ spectrum of *m*/*z* 351 (dcGTX2,3) in *M. galloprovincialis* at ESI^−^. Energy: 18 arbitrary units; Figure S8. HCD MS^2^ spectrum of *m*/*z* 351 (dcGTX2,3) in *M. galloprovincialis* at ESI^−^. Energy: 85 arbitrary units; Figure S9. CID MS^2^ spectrum of *m*/*z* 273 (dcNeo) in *M. galloprovincialis* at ESI^+^. Energy: 25 arbitrary units; Figure S10. HCD MS^2^ spectrum of *m*/*z* 273 (dcNeo) in *M. galloprovincialis* at ESI^+^. Energy: 55 arbitrary units; Figure S11. CID MS^2^ spectrum of *m*/*z* 257 (dcSTX) in *M. galloprovincialis* at ESI^+^. Energy: 30 arbitrary units; Figure S12. HCD MS^2^ spectrum of *m*/*z* 257 (dcSTX) in *M. galloprovincialis* at ESI^+^. Energy: 55 arbitrary units; Figure S13. CID MS^2^ spectrum of *m*/*z* 410 (GTX4) in *M. galloprovincialis* at ESI^−^. Energy: 25 arbitrary units; Figure S14. HCD MS^2^ spectrum of *m*/*z* 410 (GTX4) in *M. galloprovincialis* at ESI^−^. Energy: 55 arbitrary units; Figure S15. CID MS^2^ spectrum of *m*/*z* 378 (GTX5) in *M. galloprovincialis* at ESI^−^. Energy: 19 arbitrary units; Figure S16. HCD MS^2^ spectrum of *m*/*z* 378 (GTX5) in *M. galloprovincialis* at ESI^−^. Energy: 65 arbitrary units; Figure S17. CID MS^2^ spectrum of *m*/*z* 394 (GTX6) in *M. galloprovincialis* at ESI^−^. Energy: 20 arbitrary units; Figure S18. HCD MS^2^ spectrum of *m*/*z* 394 (GTX6) in *M. galloprovincialis* at ESI^−^. Energy: 55 arbitrary units; Figure S19. CID MS^2^ spectrum of *m*/*z* 316 (GTX6 [M+H-SO3]^+^ *in*-source fragmentation) in *M. galloprovincialis* at ESI^+^. Energy: 35 arbitrary units; Figure S20. HCD MS^2^ spectrum of *m*/*z* 316 (GTX6 [M+H-SO_3_]^+^ *in*-source fragmentation) in *M. galloprovincialis* at ESI^+^. Energy: 60 arbitrary units; Figure S21. CID MS^2^ spectrum of *m*/*z* 300 (STX) in *M. galloprovincialis* at ESI^+^. Energy: 20 arbitrary units; Figure S22. HCD MS^2^ spectrum of *m*/*z* 300 (STX) in *M. galloprovincialis* at ESI^+^. Energy: 55 arbitrary units; Figure S23. CID MS^2^ spectrum of *m*/*z* 396 (M1) in *M. galloprovincialis* at ESI^+^. Energy: 16 arbitrary units. Quantified in ESI^−^ *m*/*z* 394.07810; Figure S24. HCD MS^2^ spectrum of *m*/*z* 396 (M1) in *M. galloprovincialis* at ESI^+^. Energy: 70 arbitrary units. Quantified in ESI^−^ *m*/*z* 394.07810; Figure S25. CID MS^2^ spectrum of *m*/*z* 316 (M1 [M+H-SO_3_]^+^ *in*-source fragmentation) in *M. galloprovincialis* at ESI^+^. Energy: 35 arbitrary units. Quantified in ESI^−^ *m*/*z* 394.07810; Figure S26. HCD MS^2^ spectrum of *m*/*z* 316 (M1 [M+H-SO_3_]^+^ *in*-source fragmentation) in *M. galloprovincialis* at ESI^+^. Energy: 70 arbitrary units. Quantified in ESI^−^*m*/*z* 394.07810; Figure S27. CID MS^2^ spectrum of *m*/*z* 316 (M2) in *M. galloprovincialis* at ESI^+^. Energy: 35 arbitrary units; Figure S28. HCD MS^2^ spectrum of *m*/*z* 316 (M2) in *M. galloprovincialis* at ESI^+^. Energy: 60 arbitrary units; Figure S29. CID MS^2^ spectrum of *m*/*z* 316 (M6) in *M. galloprovincialis* at ESI^+^. Energy: 35 arbitrary units; Figure S30. HCD MS^2^ spectrum of *m*/*z* 316 (M6) in *M. galloprovincialis* at ESI^+^. Energy: 60 arbitrary units; Figure S31. CID MS^2^ spectrum of *m*/*z* 273 (dcM6) in *M. galloprovincialis* at ESI^+^. Energy: 35 arbitrary units; Figure S32. HCD MS^2^ spectrum of *m*/*z* 273 (dcM6) in *M. galloprovincialis* at ESI^+^. Energy: 60 arbitrary units; Figure S33. CID MS^2^ spectrum of *m*/*z* 305 (dcM10) in *M. galloprovincialis* at ESI^+^. Energy: 35 arbitrary units; Figure S34. HCD MS^2^ spectrum of *m*/*z* 305 (dcM10) in *M. galloprovincialis* at ESI^+^. Energy: 55 arbitrary units.
