# Supplementary material for: LC-HRMS Profiling of Paralytic Shellfish Toxins in Mytilus galloprovincialis after a Gymnodinium catenatum Bloom
Source: Mar Drugs. 2022 Oct 28;20(11):680. doi: 10.3390/md20110680 (PMC9694030; doi:10.3390/md20110680)
Supplement: Supplementary file 1 [file marinedrugs-20-00680-s001.zip › marinedrugs-1960349-supplementary.pdf]

Supplementary Material

LC-HRMS profiling of paralytic shellfish toxins in *Mytilus galloprovincialis* after a *Gymnodinium catenatum* bloom

Sandra Lage [1,*], Pedro Reis Costa [1,2], Adelino V. M. Canário [1], José P. Da Silva [1]

[1] Centre of Marine Sciences (CCMAR/CIMAR LA), University of Algarve, Campus de Gambelas, 8005-139, Faro, Portugal;

[2] Portuguese Institute for the Sea and Atmosphere (IPMA), Av. Brasília, 1449-006 Lisbon, Portugal

[*] Correspondence: smlage@ualg.pt (S.L.)

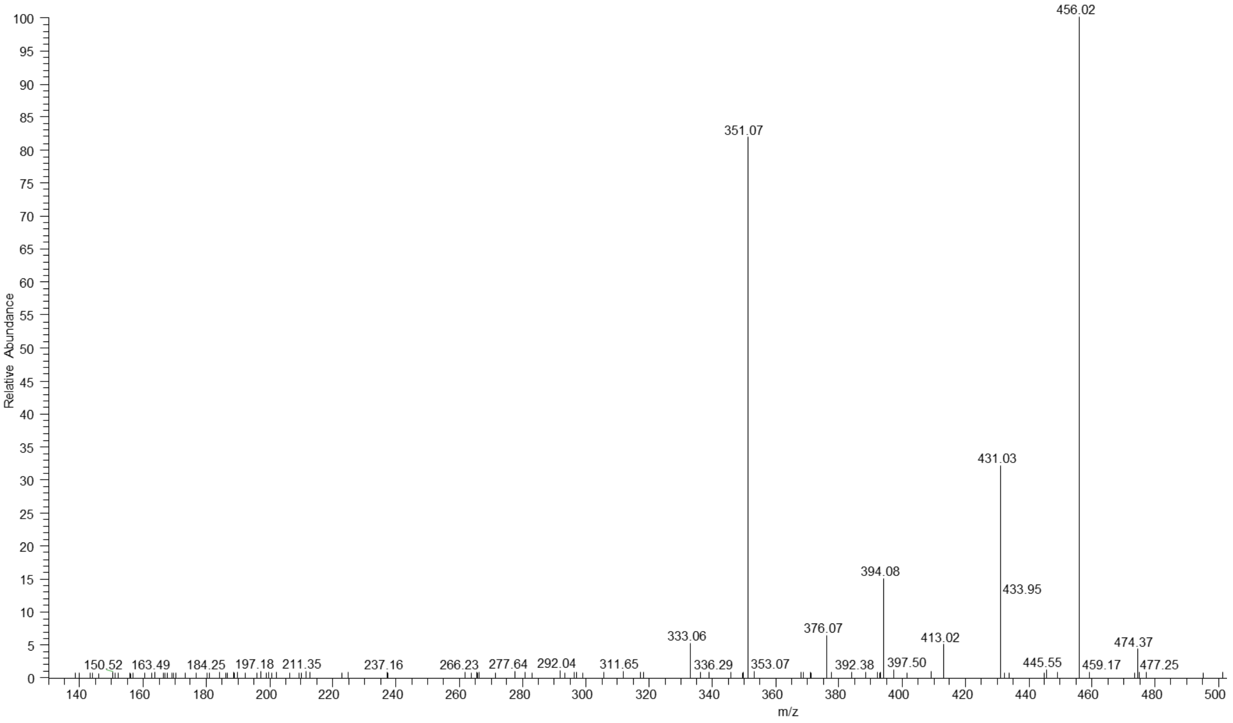

Figure S1. CID MS^2 spectrum of *m/z* 474 (C1/2) in *M. galloprovincialis* at ESI^-. Energy: 25 arbitrary units.

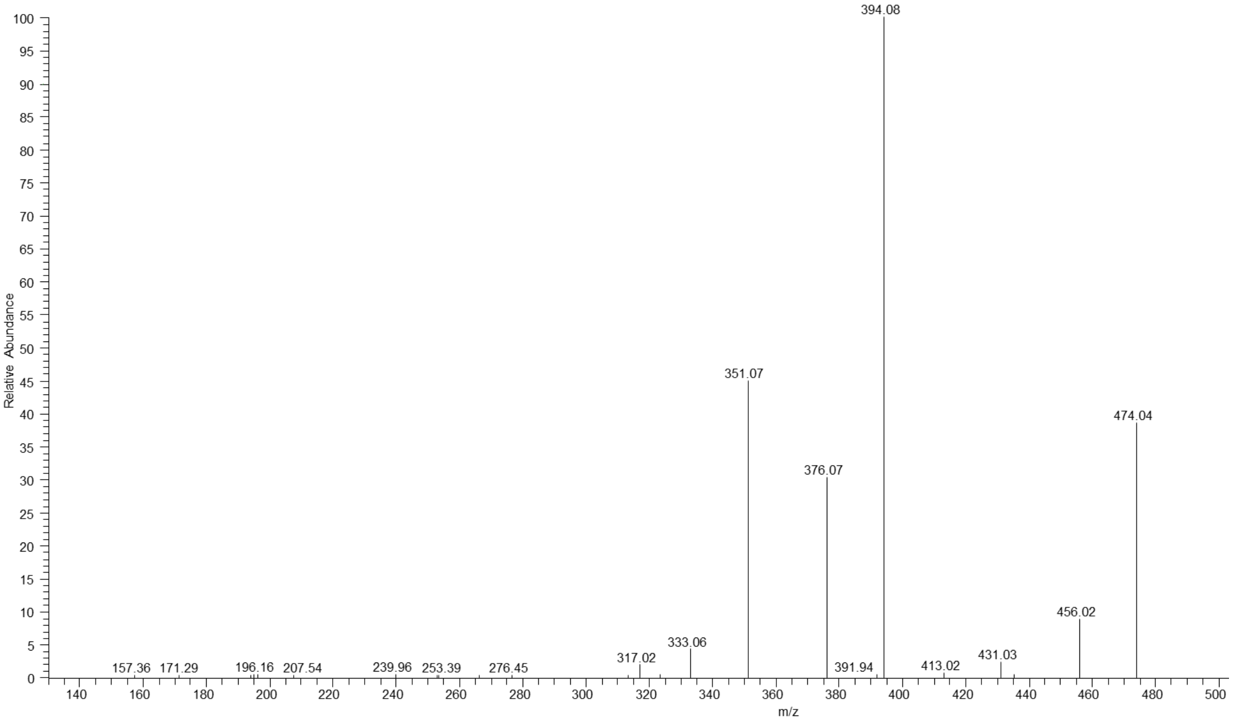

Figure S2. HCD MS^2 spectrum of *m/z* 474 (C1/2) in *M. galloprovincialis* at ESI^-. Energy: 55 arbitrary units.

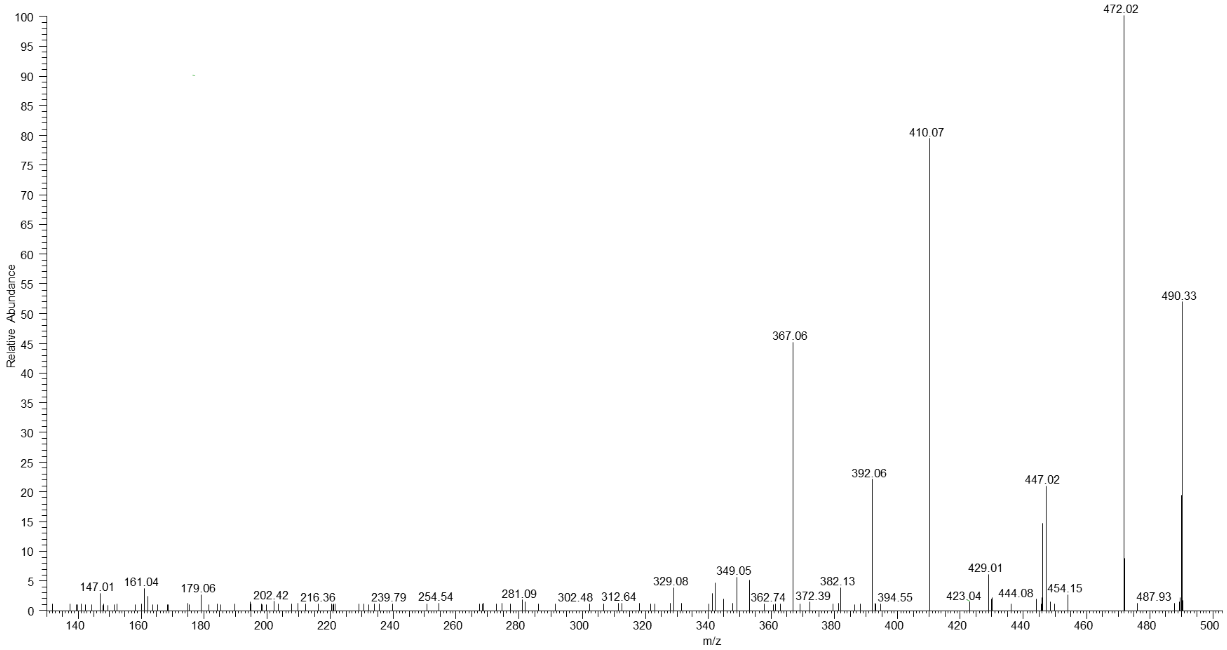

Figure S3. CID MS^2 spectrum of *m/z* 490 (C3/4) in *M. galloprovincialis* at ESI$^-$. Energy: 18 arbitrary units.

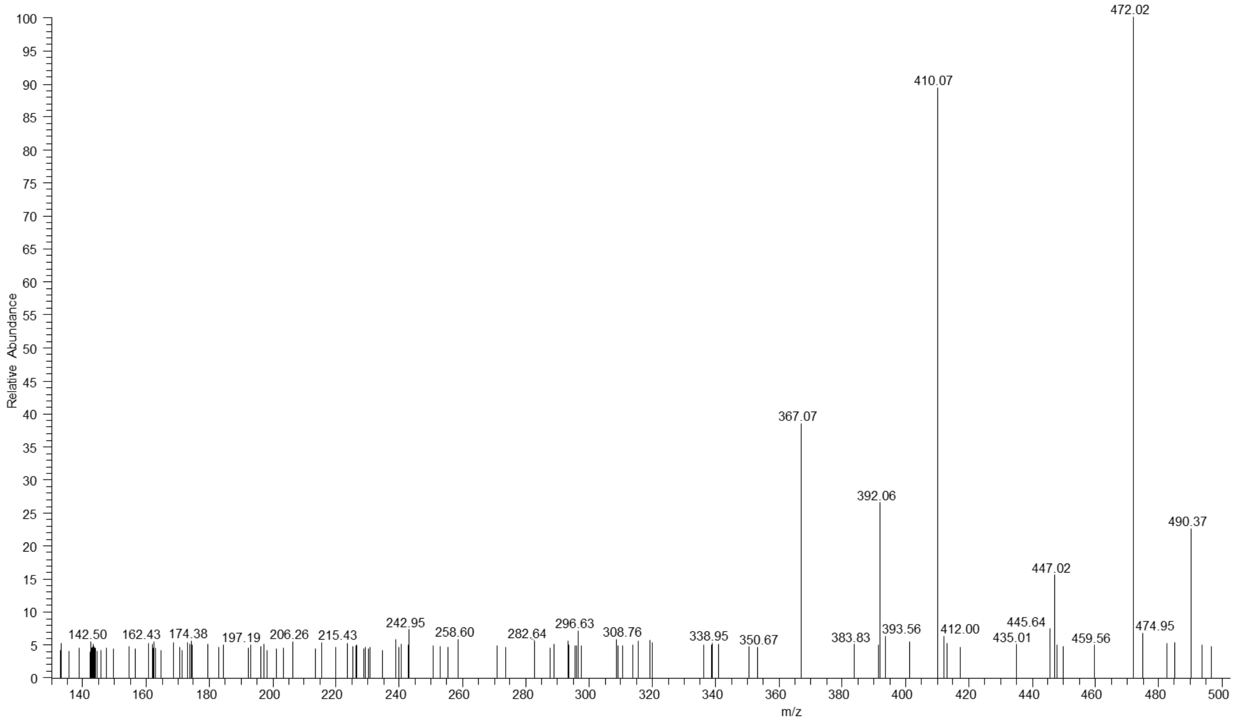

Figure S4. HCD MS^2 spectrum of *m/z* 490 (C3/4) in *M. galloprovincialis* at ESI$^-$. Energy: 55 arbitrary units.

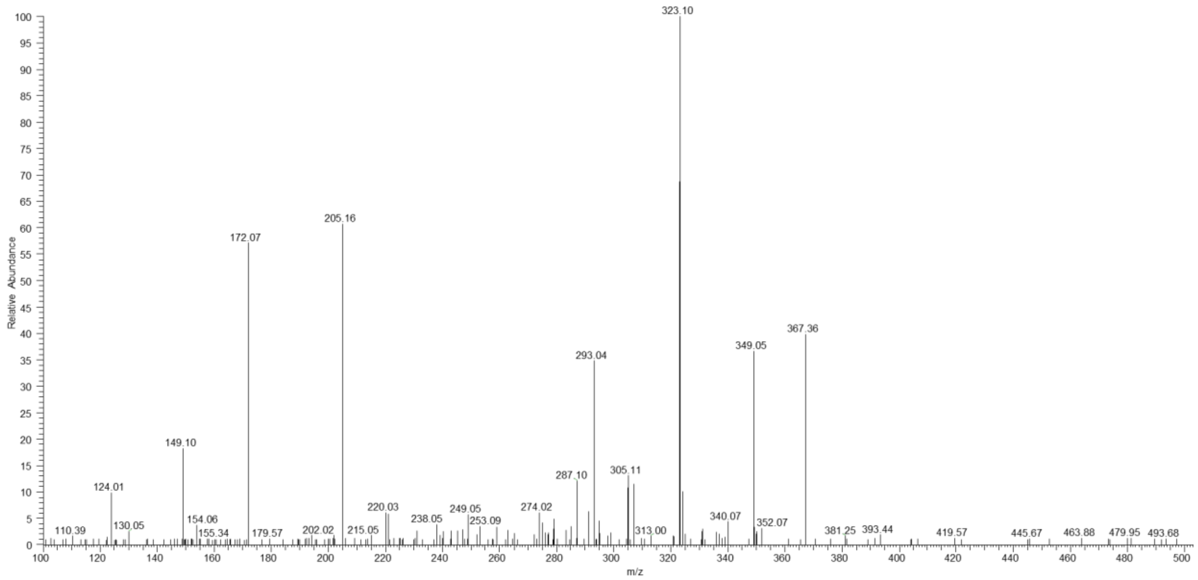

Figure S5. CID MS^2 spectrum of *m/z* 367 (dcGTX1/4) in *M. galloprovincialis* at ESI^-. Energy: 35 arbitrary units.

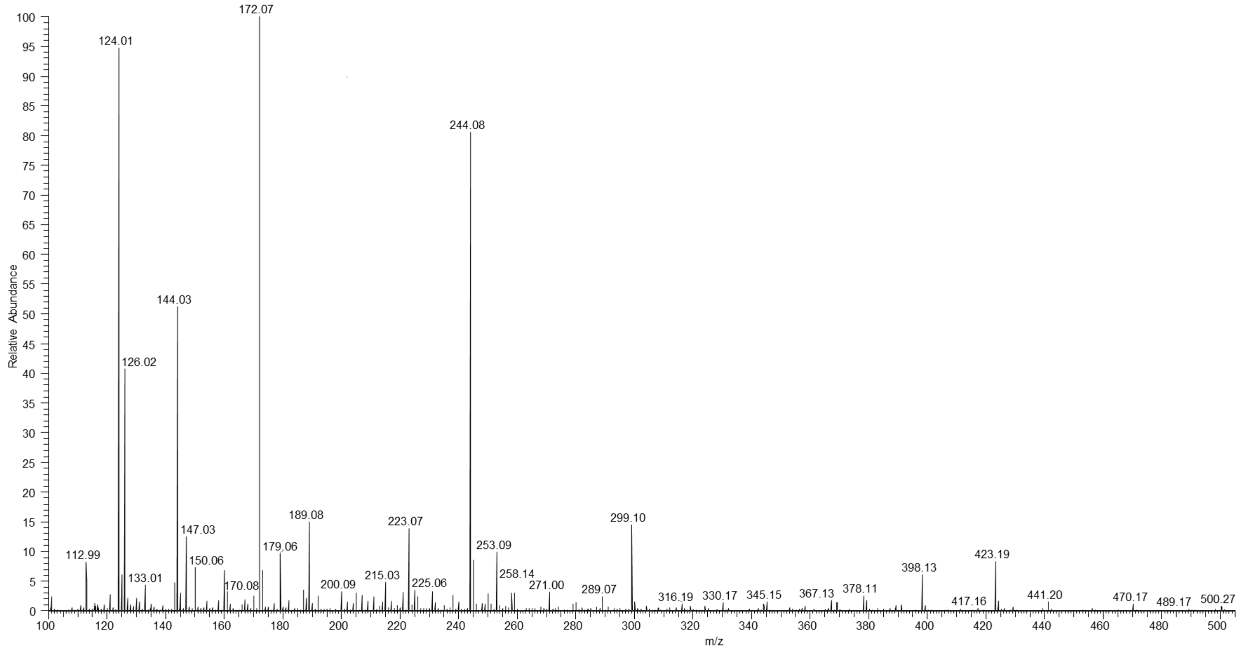

Figure S6. HCD MS^2 spectrum of *m/z* 367 (dcGTX1/4) in *M. galloprovincialis* at ESI^-. Energy: 55 arbitrary units.

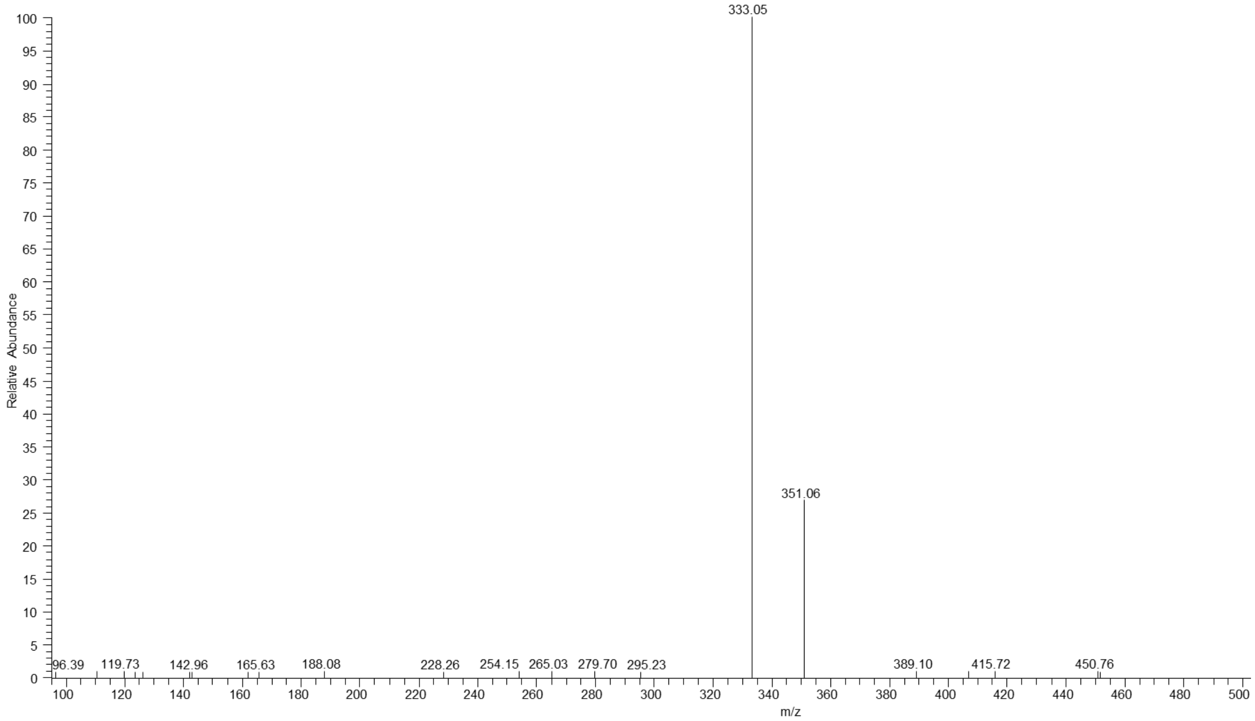

Figure S7. CID MS^2 spectrum of *m/z* 351 (dcGTX2/3) in *M. galloprovincialis* at ESI^-. Energy: 18 arbitrary units.

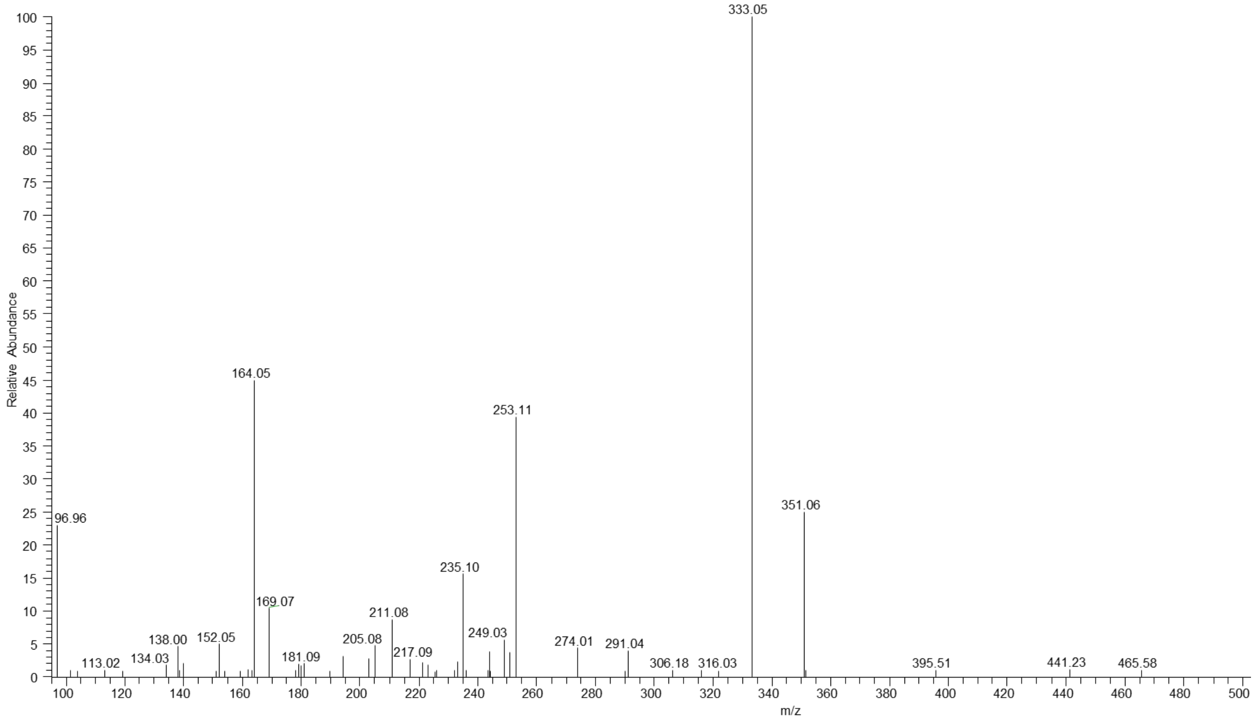

Figure S8. HCD MS^2 spectrum of *m/z* 351 (dcGTX2/3) in *M. galloprovincialis* at ESI^-. Energy: 85 arbitrary units.

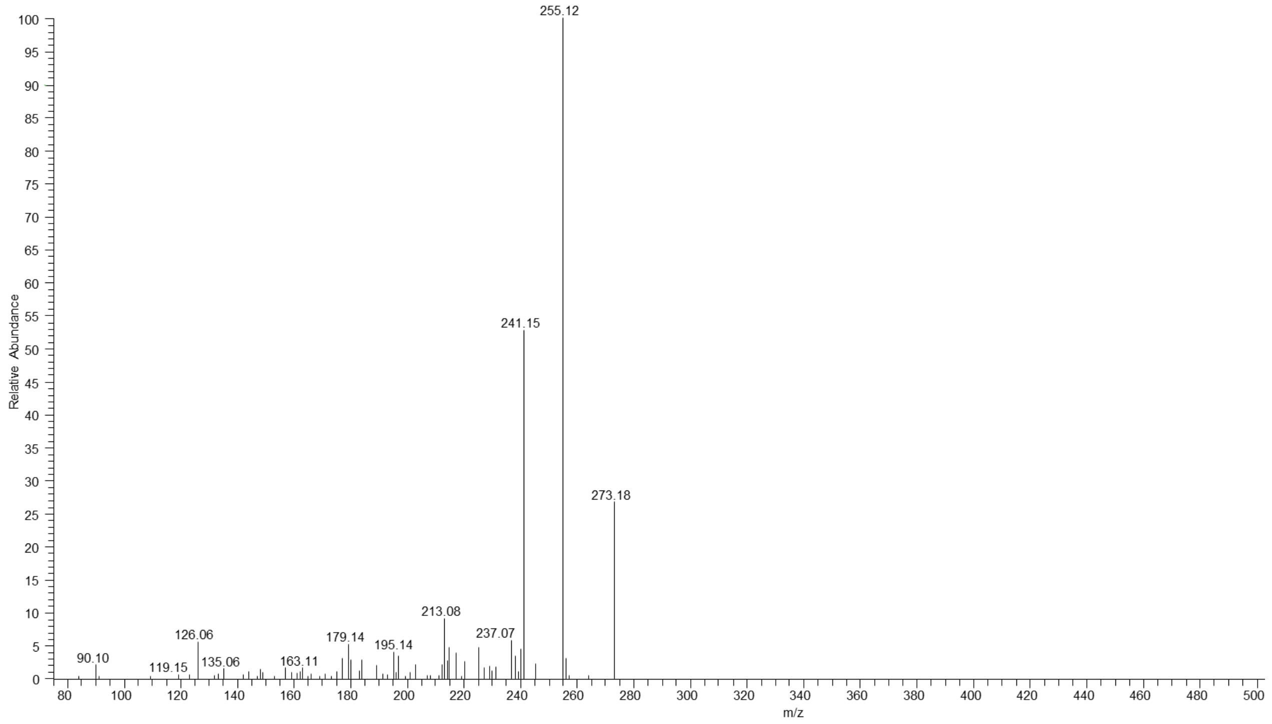

Figure S9. CID MS^2 spectrum of *m/z* 273 (dcNeo) in *M. galloprovincialis* at ESI^+. Energy: 25 arbitrary units.

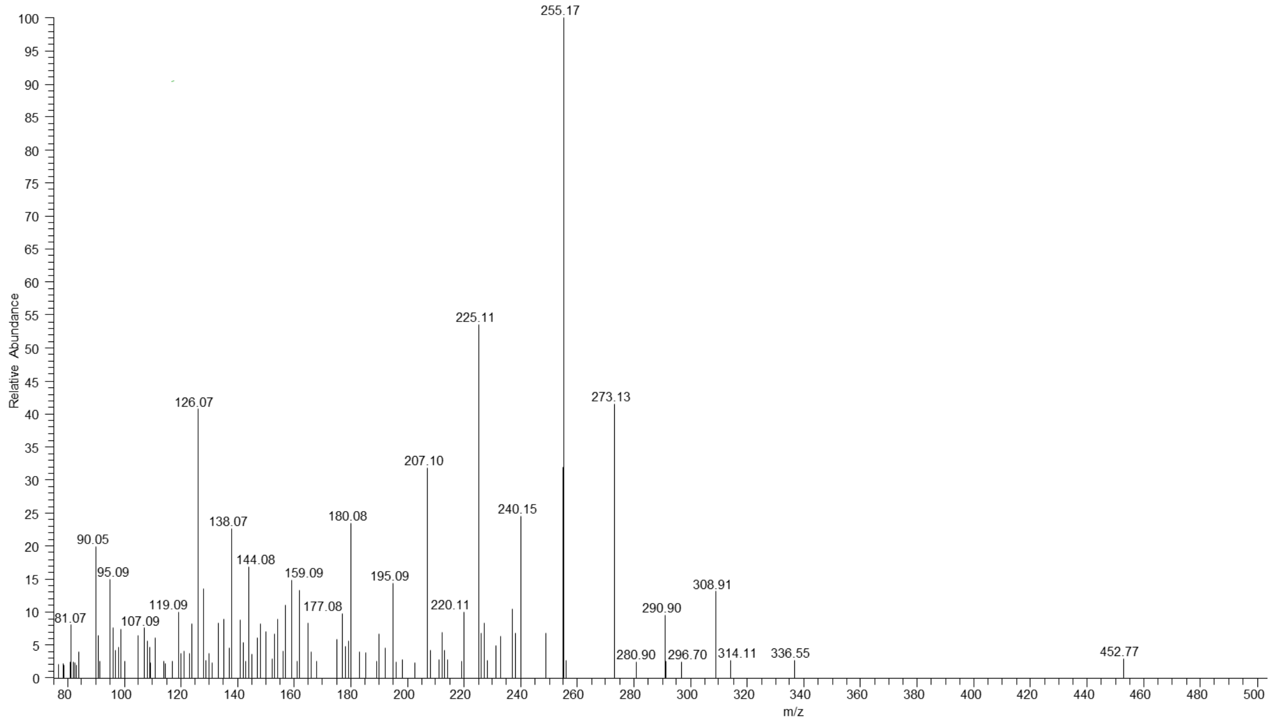

Figure S10. HCD MS^2 spectrum of *m/z* 273 (dcNeo) in *M. galloprovincialis* at ESI^+. Energy: 55 arbitrary units.

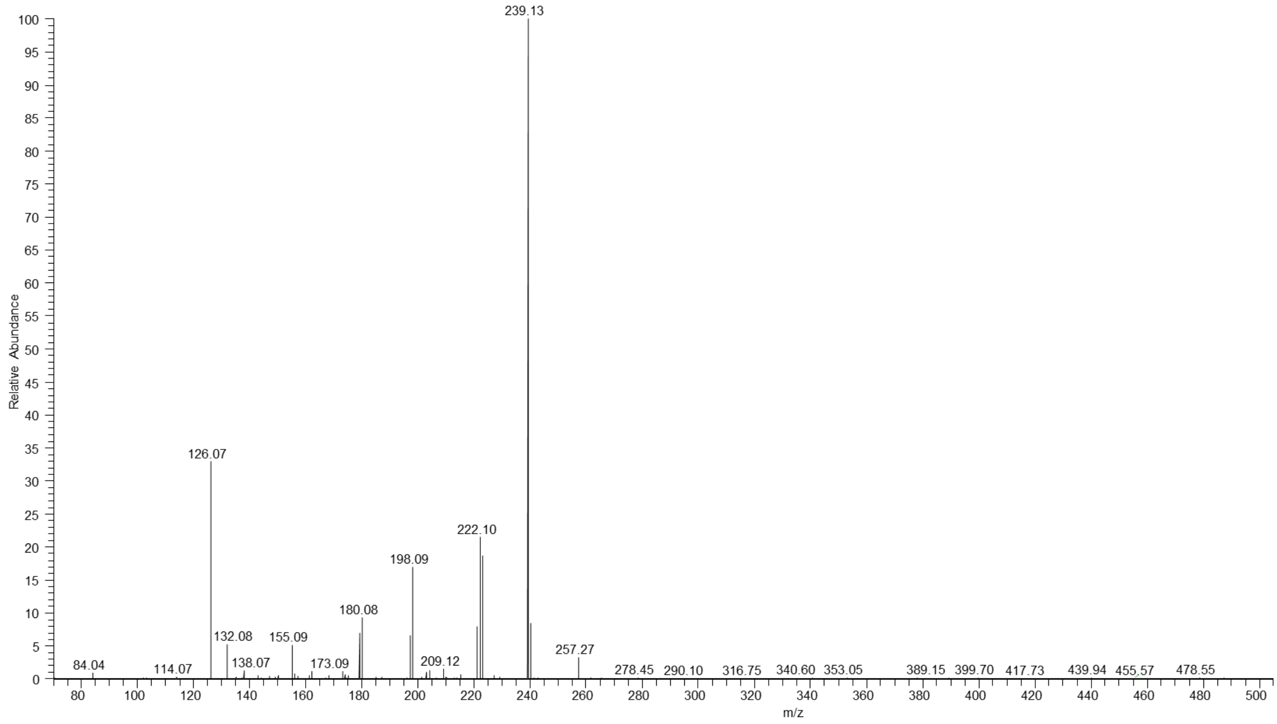

Figure S11. CID MS^2 spectrum of *m/z* 257 (dcSTX) in *M. galloprovincialis* at ESI^+. Energy: 30 arbitrary units.

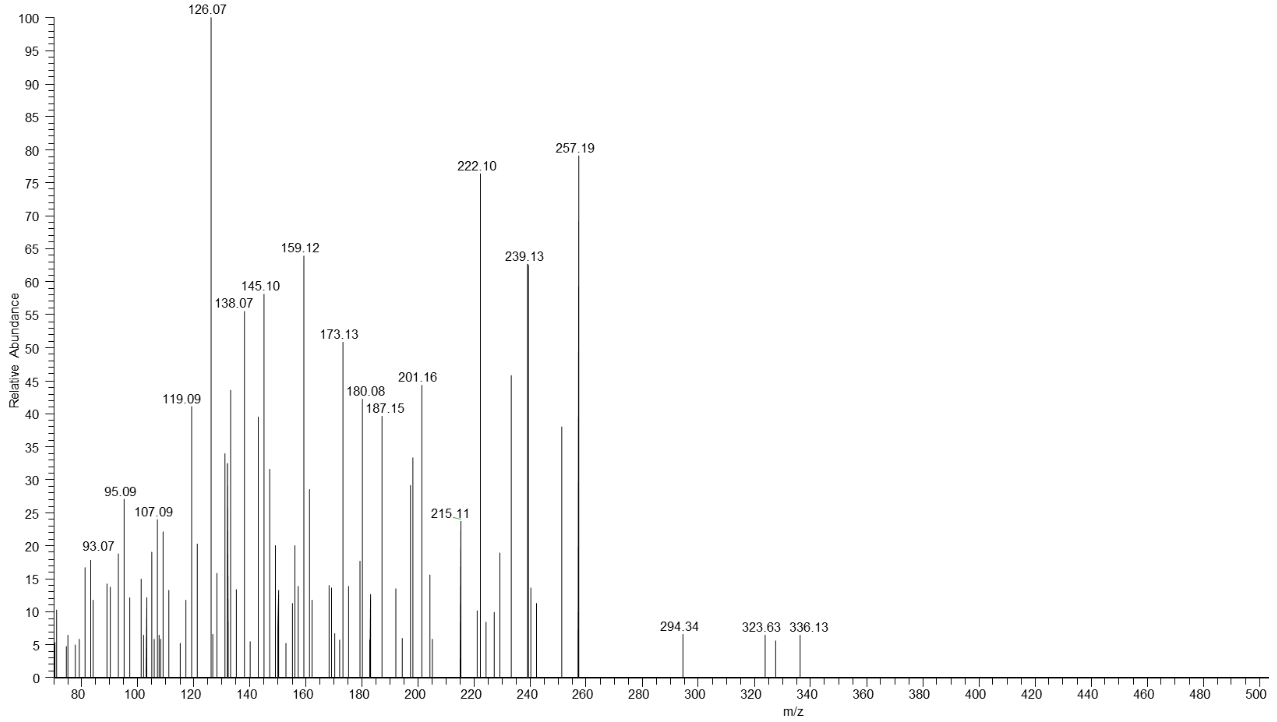

Figure S12. HCD MS^2 spectrum of *m/z* 257 (dcSTX) in *M. galloprovincialis* at ESI^+. Energy: 55 arbitrary units.

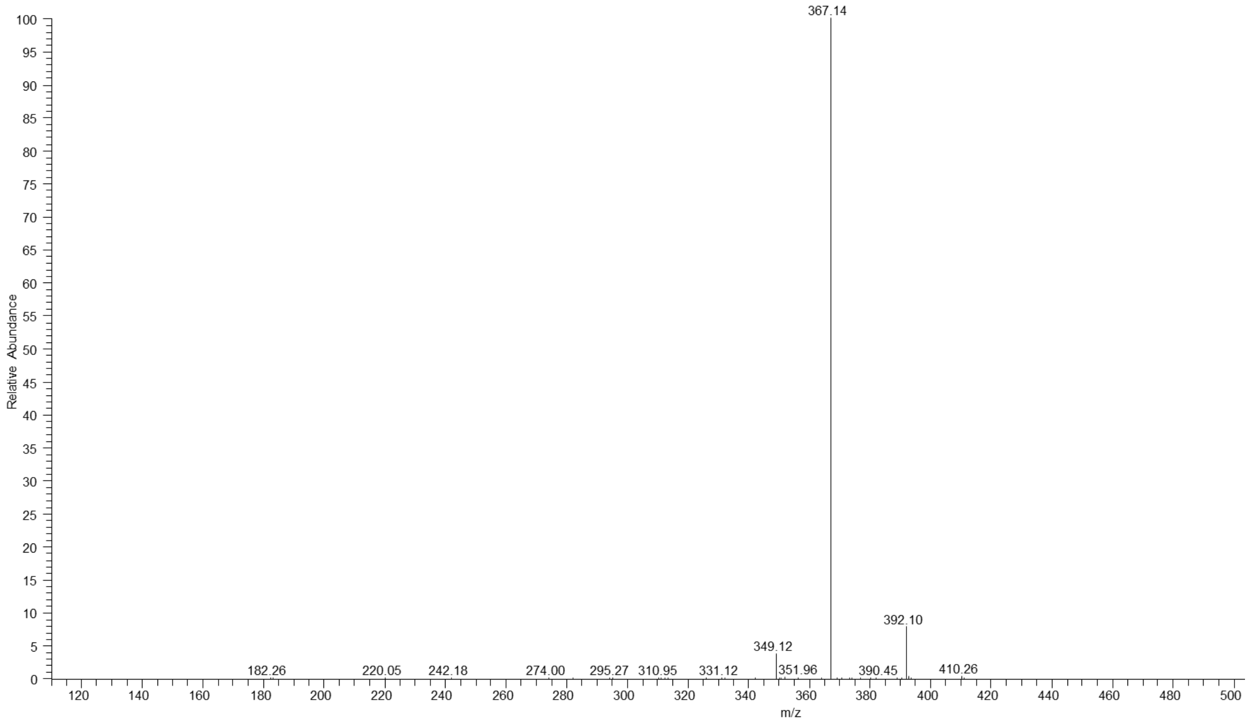

Figure S13. CID MS^2 spectrum of *m/z* 410 (GTX4) in *M. galloprovincialis* at ESI^-. Energy: 25 arbitrary units.

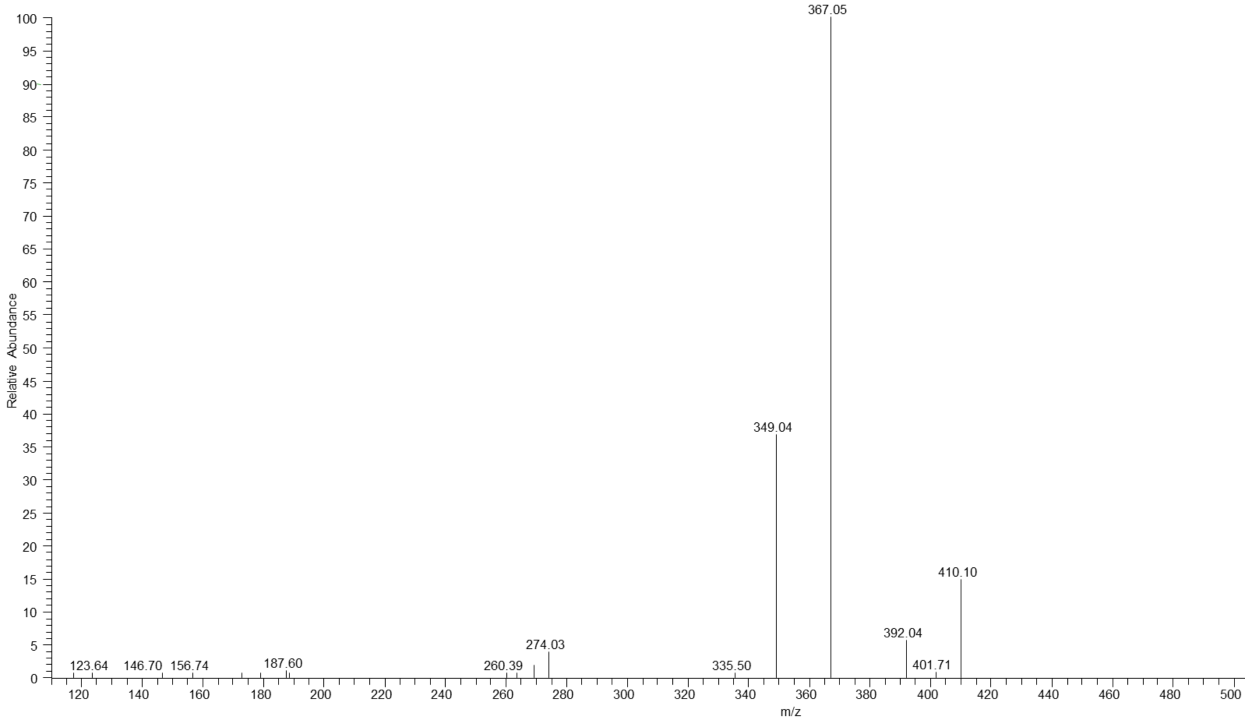

Figure S14. HCD MS^2 spectrum of *m/z* 410 (GTX4) in *M. galloprovincialis* at ESI^-. Energy: 55 arbitrary units.

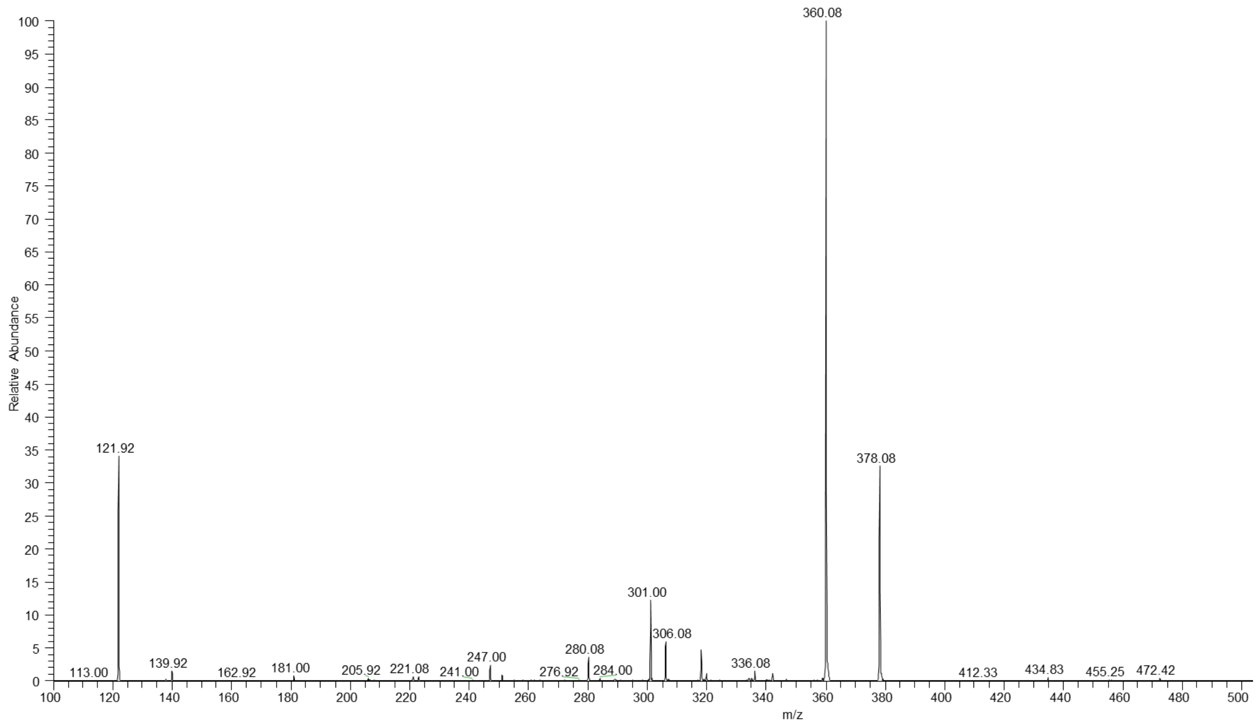

Figure S15. CID MS^2 spectrum of *m/z* 378 (GTX5) in *M. galloprovincialis* at ESI^-. Energy: 19 arbitrary units.

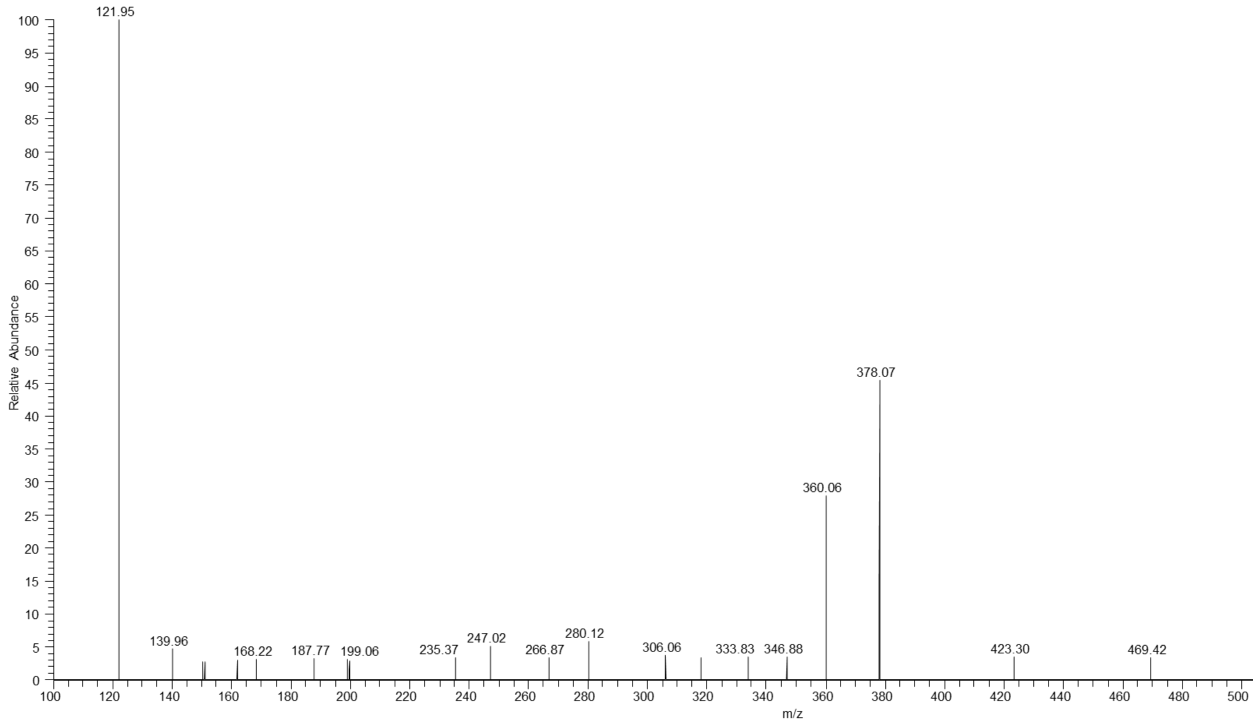

Figure S16. HCD MS^2 spectrum of *m/z* 378 (GTX5) in *M. galloprovincialis* at ESI^-. Energy: 65 arbitrary units.

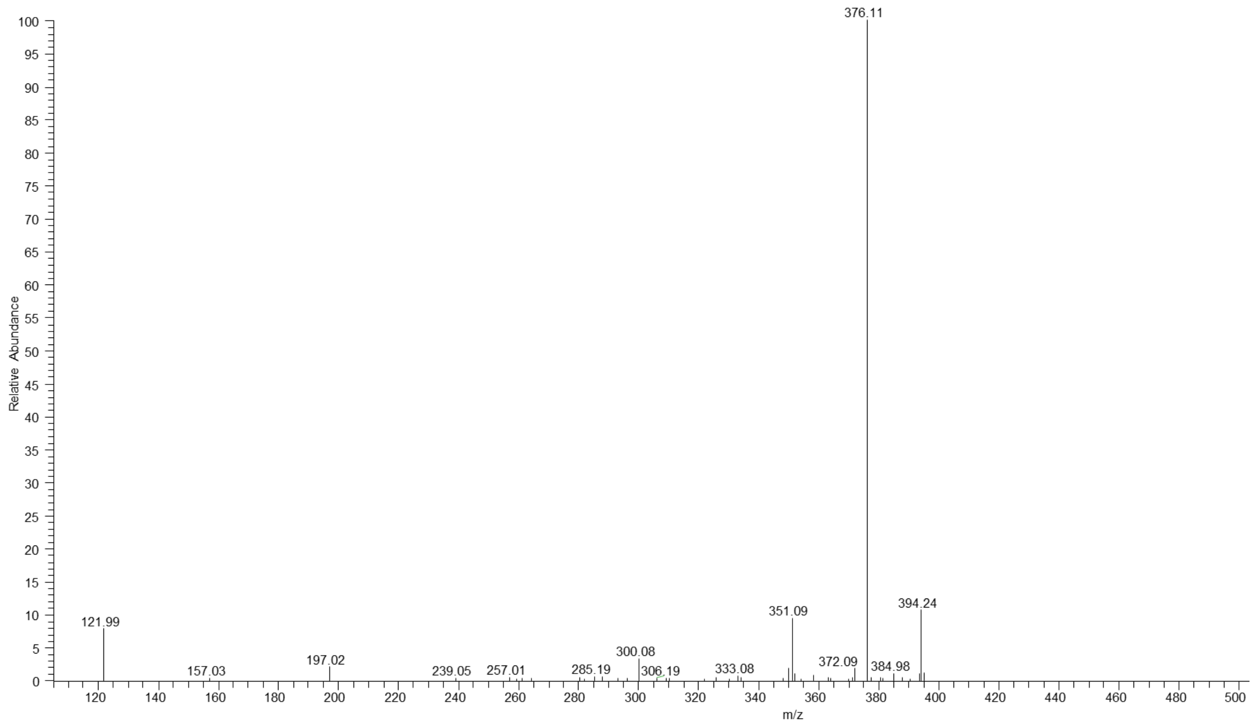

Figure S17. CID MS^2 spectrum of *m/z* 394 (GTX6) in *M. galloprovincialis* at ESI^-. Energy: 20 arbitrary units.

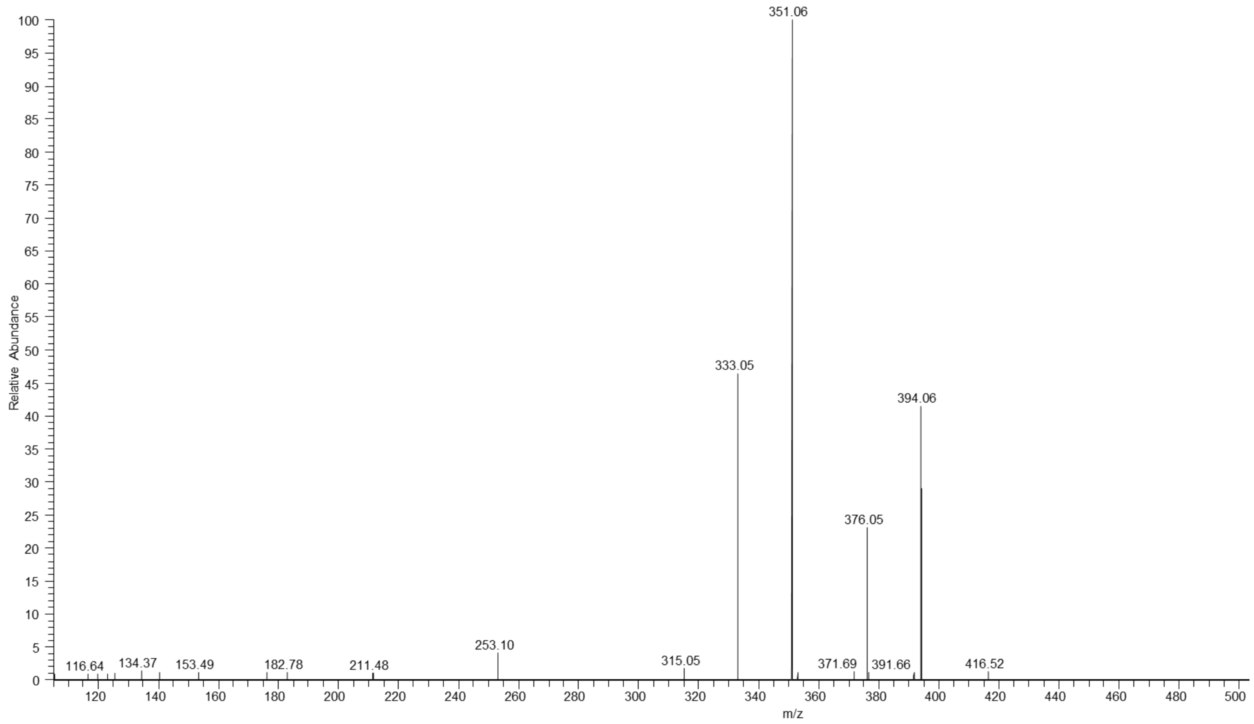

Figure S18. HCD MS^2 spectrum of *m/z* 394 (GTX6) in *M. galloprovincialis* at ESI^-. Energy: 55 arbitrary units.

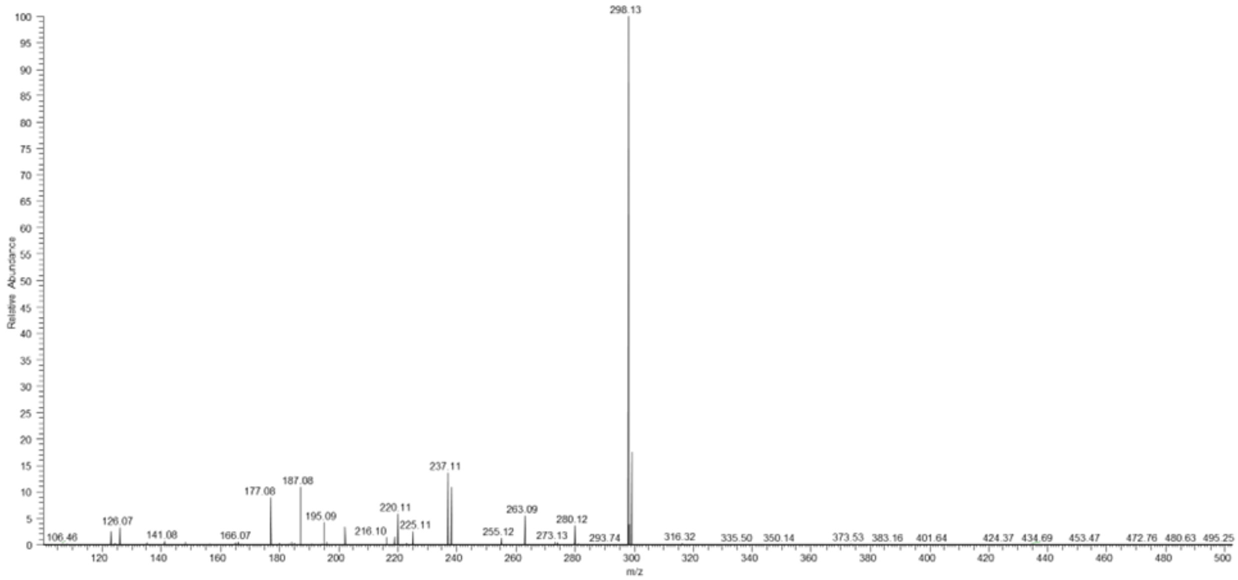

Figure S19. CID MS^2 spectrum of *m/z* 316 (GTX6 $[M + H – SO_3]^+$ *in*-source fragmentation) in *M. galloprovincialis* at ESI^+. Energy: 35 arbitrary units.

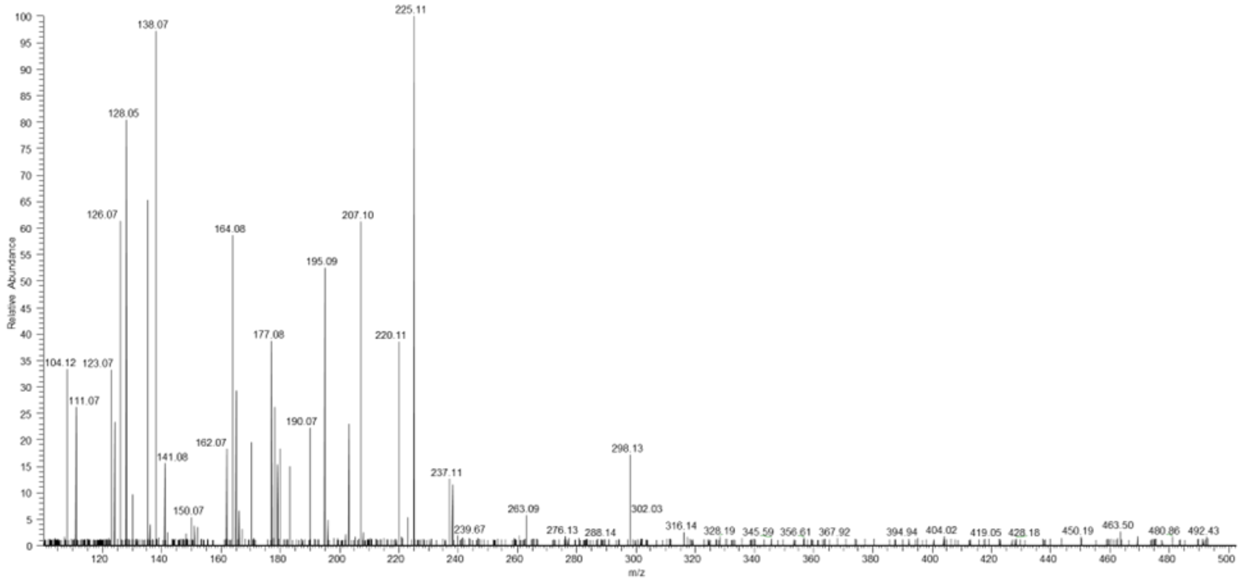

Figure S20. HCD MS^2 spectrum of *m/z* 316 (GTX6 $[M + H – SO_3]^+$ *in*-source fragmentation) in *M. galloprovincialis* at ESI^+. Energy: 60 arbitrary units.

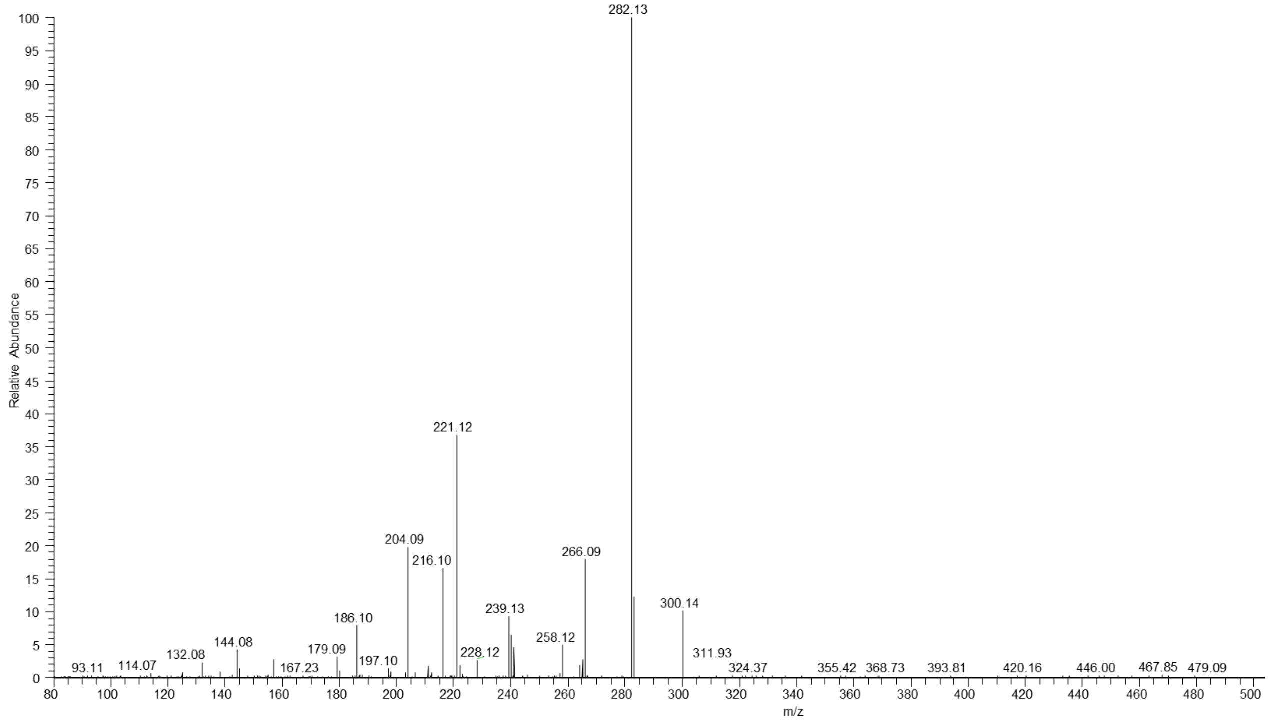

Figure S21. CID MS^2 spectrum of *m/z* 300 (STX) in *M. galloprovincialis* at ESI^+. Energy: 20 arbitrary units.

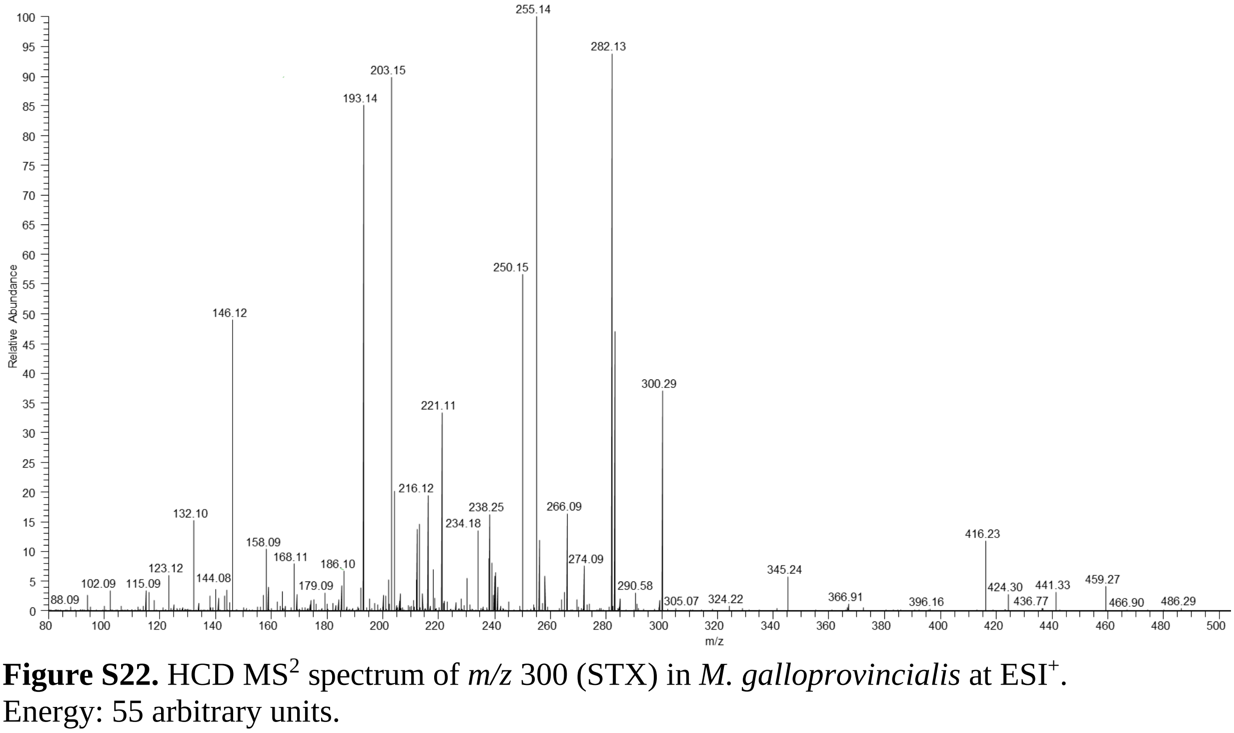

Figure S22. HCD MS^2 spectrum of *m/z* 300 (STX) in *M. galloprovincialis* at ESI^+. Energy: 55 arbitrary units.

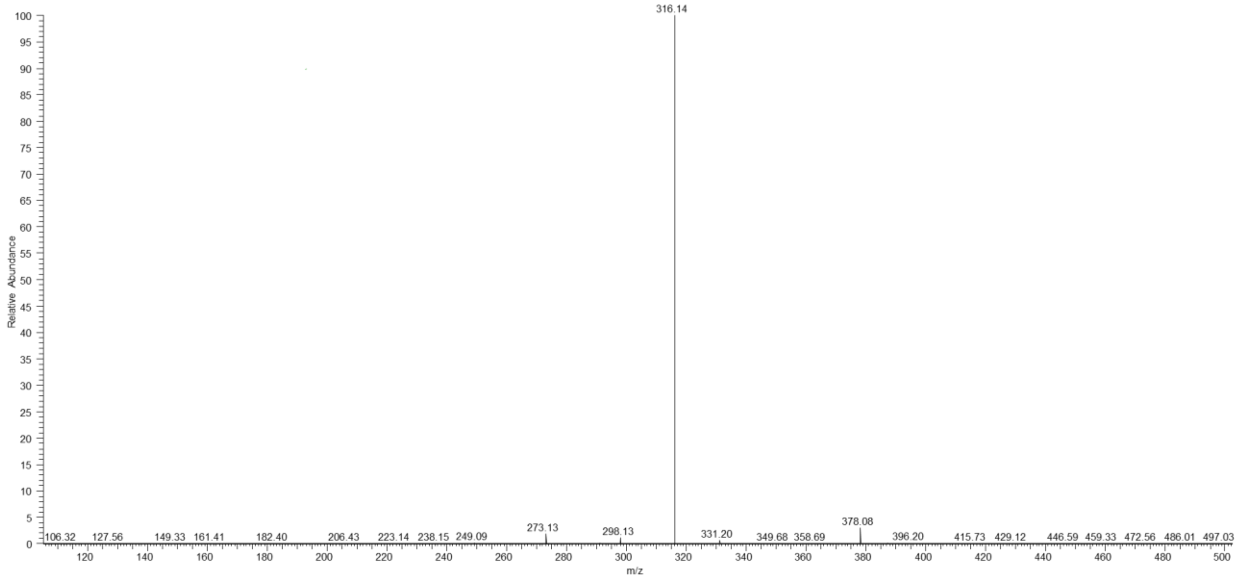

Figure S23. CID MS^2 spectrum of *m/z* 396 (M1) in *M. galloprovincialis* at ESI^+. Energy: 16 arbitrary units. Quantified in ESI^- *m/z* 394.07810.

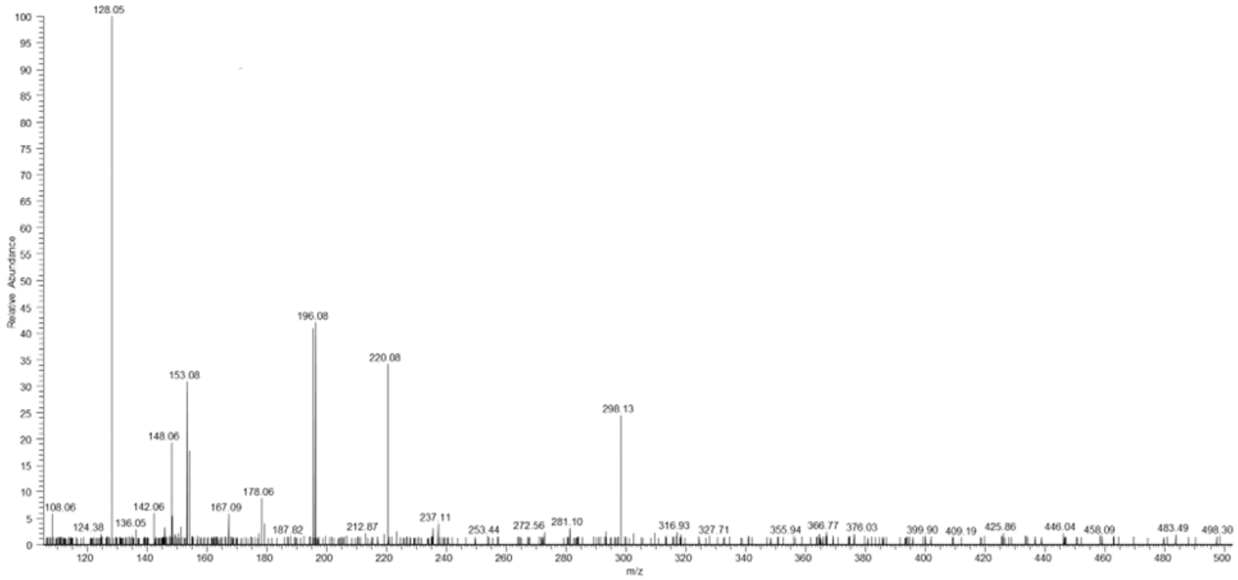

Figure S24. HCD MS^2 spectrum of *m/z* 396 (M1) in *M. galloprovincialis* at ESI^+. Energy: 70 arbitrary units. Quantified in ESI^- *m/z* 394.07810.

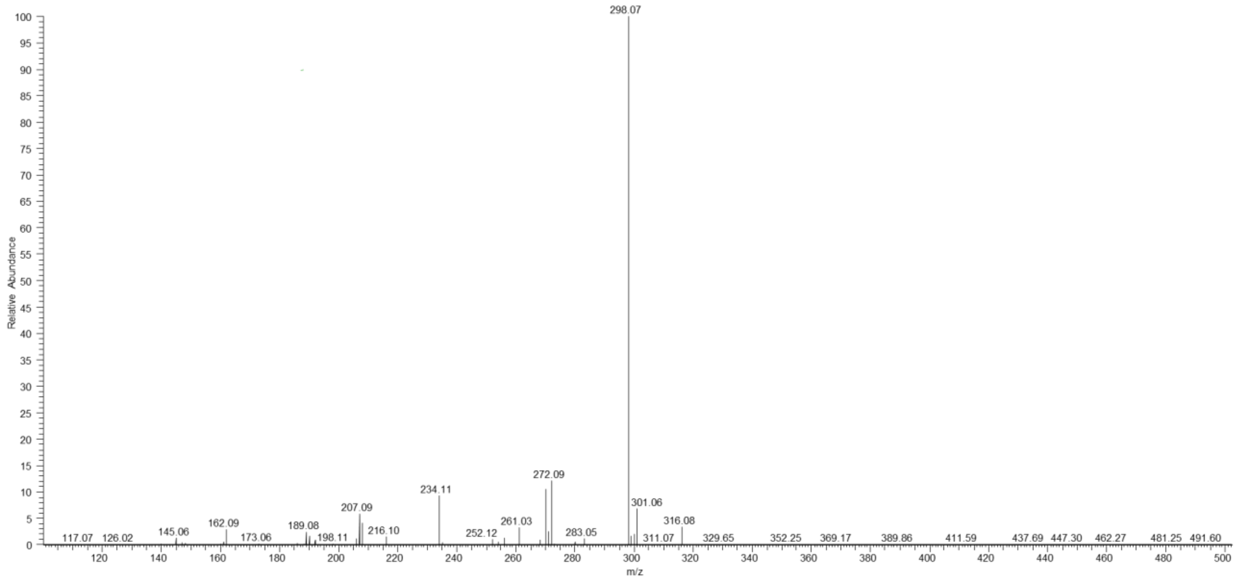

Figure S25. CID MS^2 spectrum of *m/z* 316 (M1 $[M + H - SO_3]^+$ *in*-source fragmentation) in *M. galloprovincialis* at ESI^+. Energy: 35 arbitrary units. Quantified in ESI^- *m/z* 394.07810.

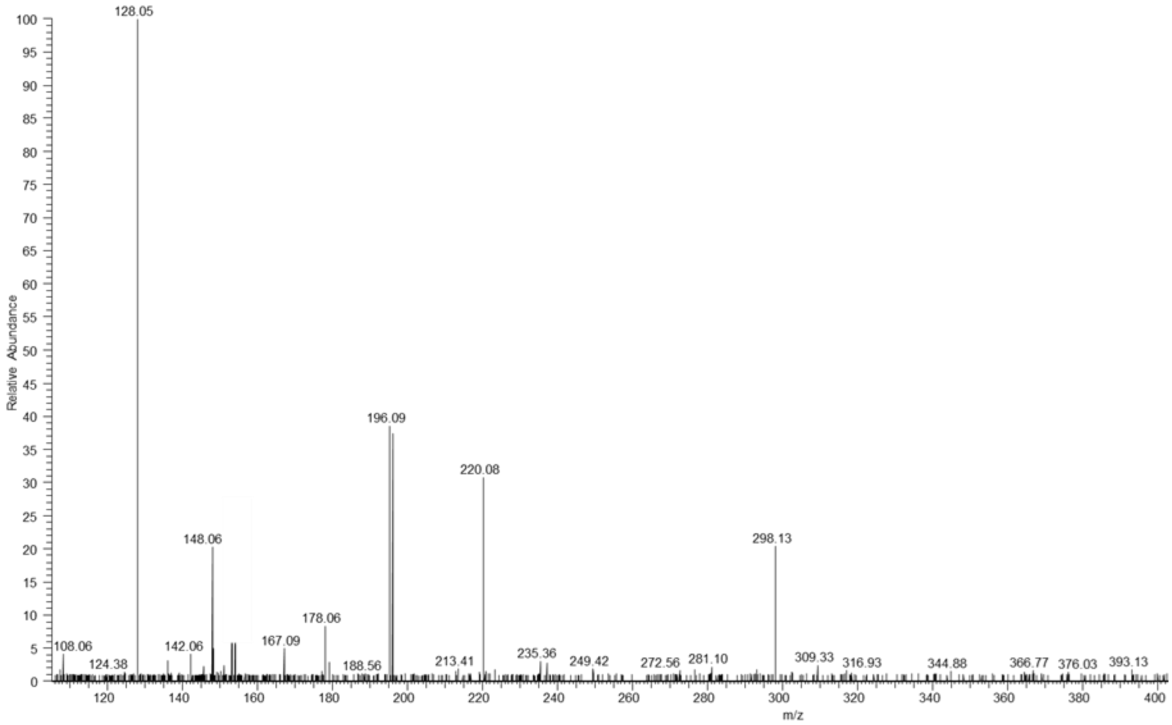

Figure S26. HCD MS^2 spectrum of *m/z* 316 (M1 $[M + H - SO_3]^+$ *in*-source fragmentation) in *M. galloprovincialis* at ESI^+. Energy: 70 arbitrary units. Quantified in ESI^- *m/z* 394.07810.

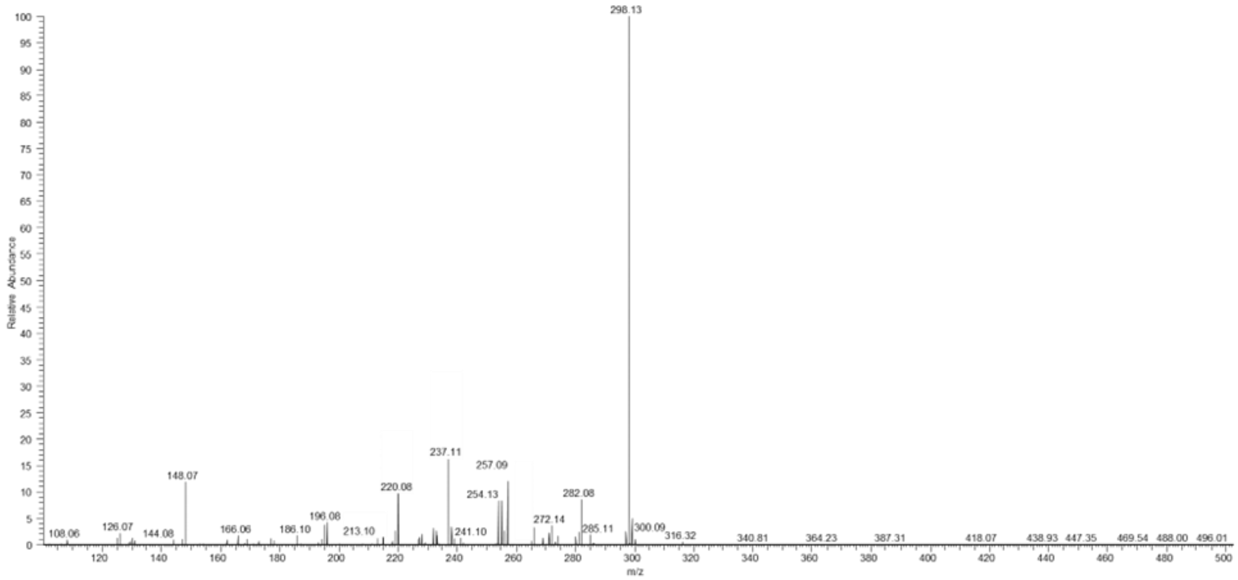

Figure S27. CID MS^2 spectrum of *m/z* 316 (M2) in *M. galloprovincialis* at ESI^+.
Energy: 35 arbitrary units.

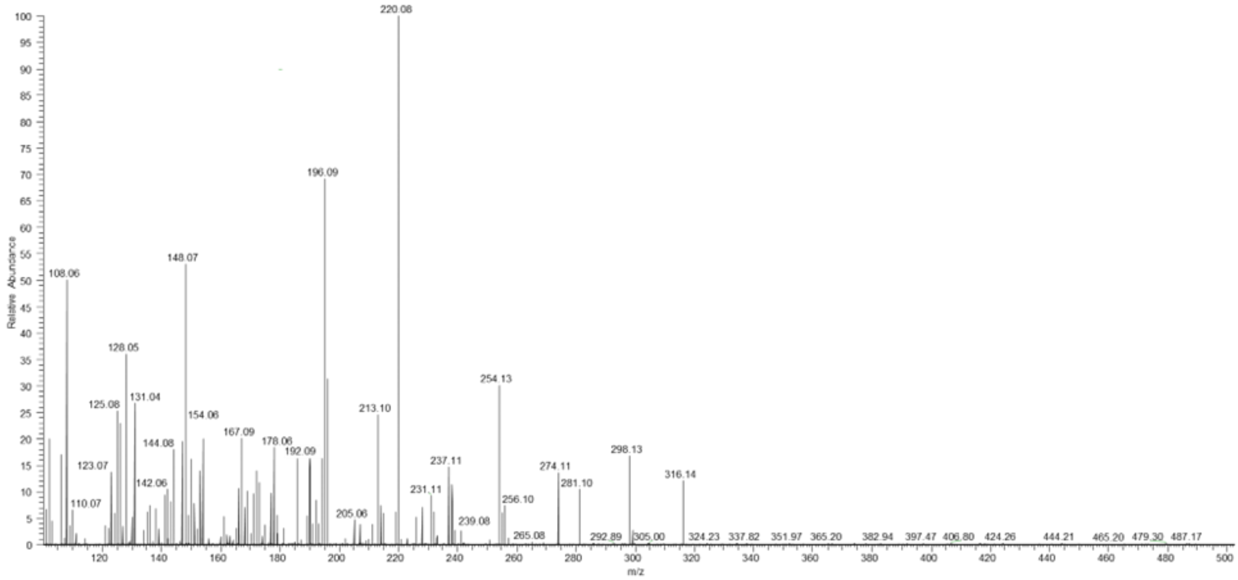

Figure S28. HCD MS^2 spectrum of *m/z* 316 (M2) in *M. galloprovincialis* at ESI^+.
Energy: 60 arbitrary units.

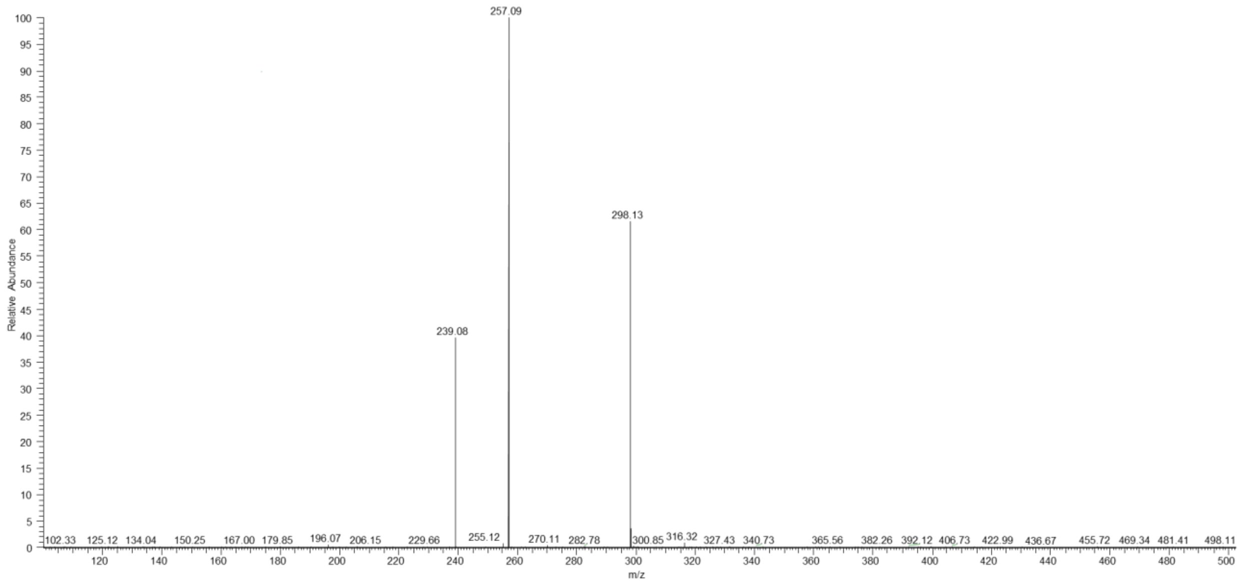

Figure S29. CID MS^2 spectrum of *m/z* 316 (M6) in *M. galloprovincialis* at ESI^+.
Energy: 35 arbitrary units.

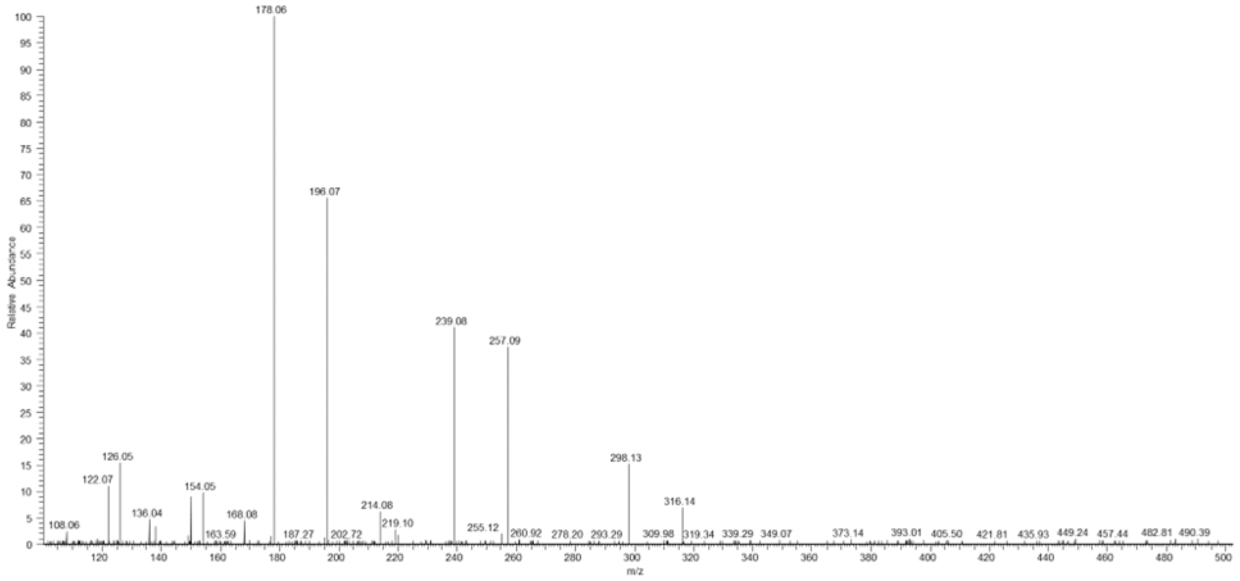

Figure S30. HCD MS^2 spectrum of *m/z* 316 (M6) in *M. galloprovincialis* at ESI^+.
Energy: 60 arbitrary units.

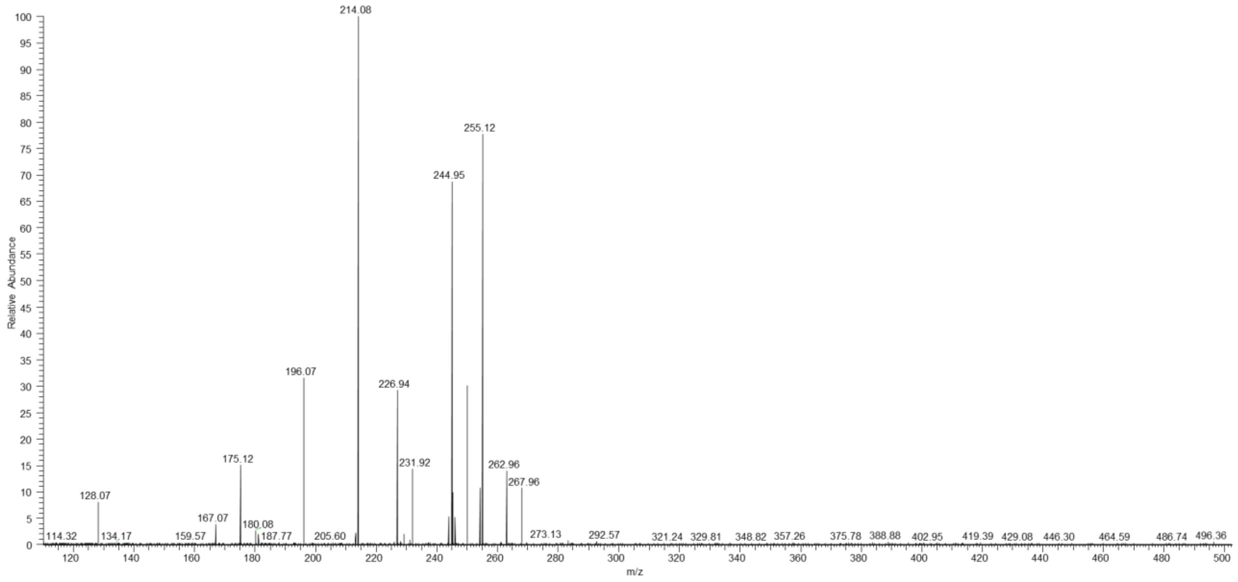

Figure S31. CID MS^2 spectrum of *m/z* 273 (dcM6) in *M. galloprovincialis* at ESI$^+$. Energy: 35 arbitrary units.

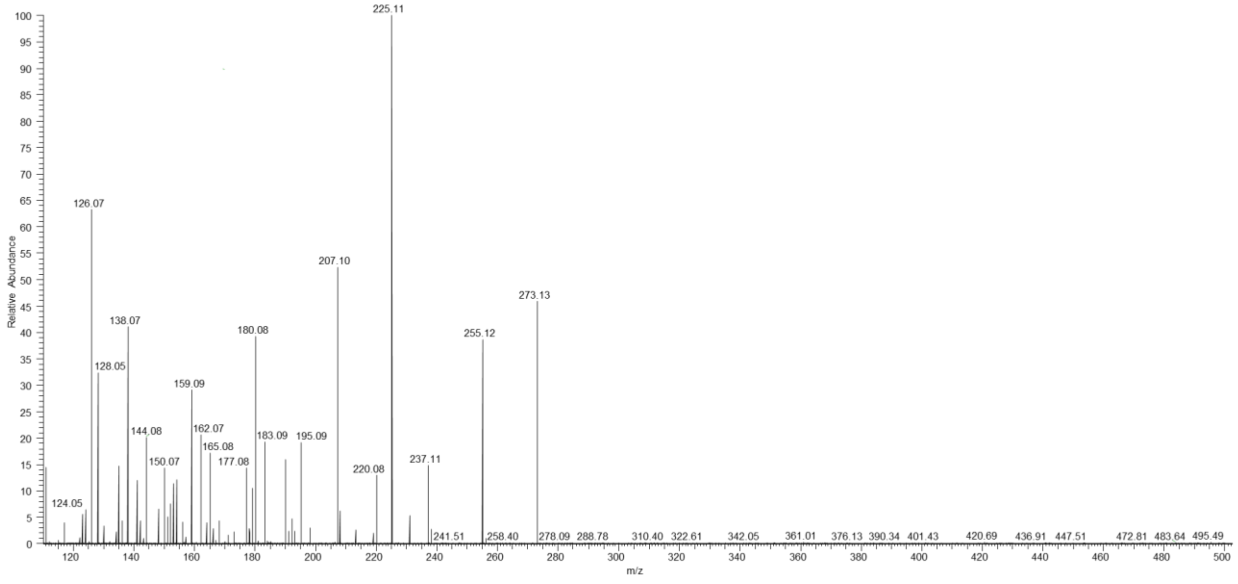

Figure S32. HCD MS^2 spectrum of *m/z* 273 (dcM6) in *M. galloprovincialis* at ESI$^+$. Energy: 60 arbitrary units.

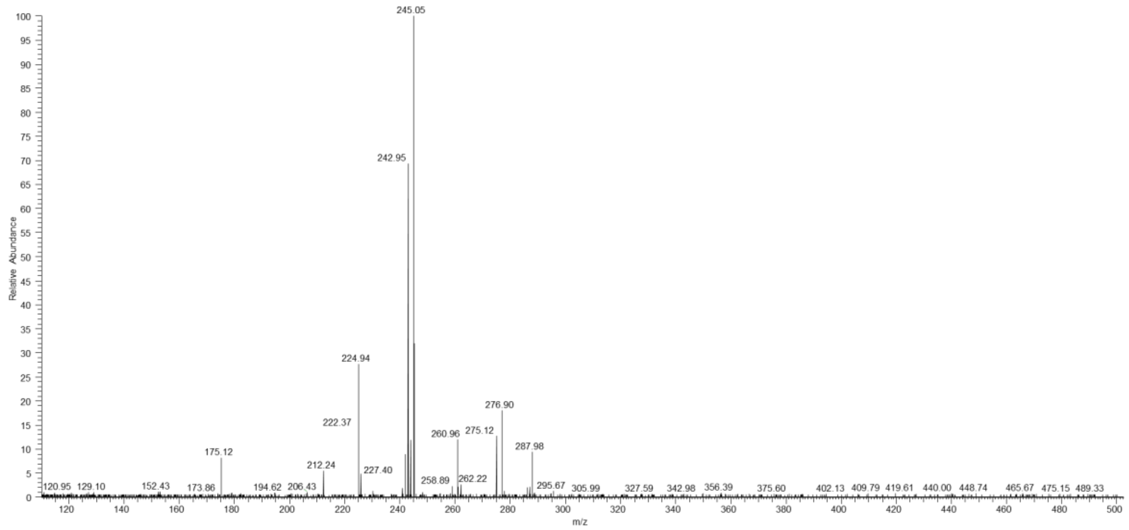

Figure S33. CID MS^2 spectrum of *m/z* 305 (dcM10) in *M. galloprovincialis* at ESI^+. Energy: 35 arbitrary units.

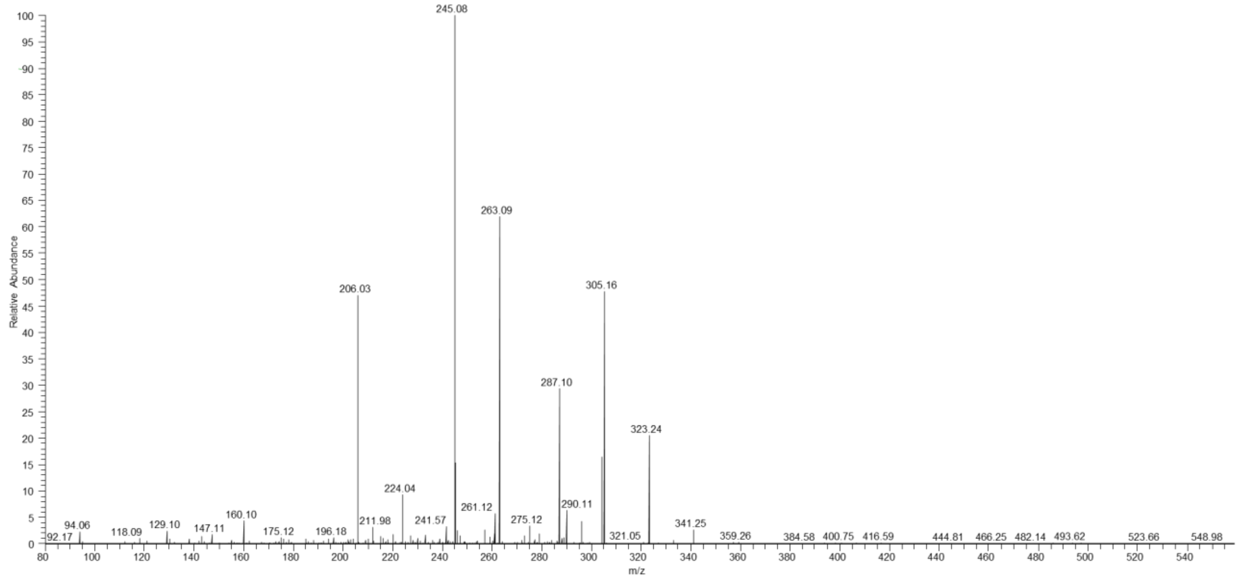

Figure S34. HCD MS^2 spectrum of *m/z* 305 (dcM10) in *M. galloprovincialis* at ESI^+. Energy: 55 arbitrary units.
